# Development of a new version of the Liverpool Malaria Model. I. Refining the parameter settings and mathematical formulation of basic processes based on a literature review

**DOI:** 10.1186/1475-2875-10-35

**Published:** 2011-02-11

**Authors:** Volker Ermert, Andreas H Fink, Anne E Jones, Andrew P Morse

**Affiliations:** 1 Institute of Geophysics and Meteorology, University of Cologne, Cologne, Germany; 2 School of Environmental Sciences, University of Liverpool, Liverpool, UK

## Abstract

**Background:**

A warm and humid climate triggers several water-associated diseases such as malaria. Climate- or weather-driven malaria models, therefore, allow for a better understanding of malaria transmission dynamics. The *Liverpool Malaria Model *(LMM) is a mathematical-biological model of malaria parasite dynamics using daily temperature and precipitation data. In this study, the parameter settings of the LMM are refined and a new mathematical formulation of key processes related to the growth and size of the vector population are developed.

**Methods:**

One of the most comprehensive studies to date in terms of gathering entomological and parasitological information from the literature was undertaken for the development of a new version of an existing malaria model. The knowledge was needed to allow the justification of new settings of various model parameters and motivated changes of the mathematical formulation of the LMM.

**Results:**

The first part of the present study developed an improved set of parameter settings and mathematical formulation of the LMM. Important modules of the original LMM version were enhanced in order to achieve a higher biological and physical accuracy. The oviposition as well as the survival of immature mosquitoes were adjusted to field conditions via the application of a fuzzy distribution model. Key model parameters, including the mature age of mosquitoes, the survival probability of adult mosquitoes, the human blood index, the mosquito-to-human (human-to-mosquito) transmission efficiency, the human infectious age, the recovery rate, as well as the gametocyte prevalence, were reassessed by means of entomological and parasitological observations. This paper also revealed that various malaria variables lack information from field studies to be set properly in a malaria modelling approach.

**Conclusions:**

Due to the multitude of model parameters and the uncertainty involved in the setting of parameters, an extensive literature survey was carried out, in order to produce a refined set of settings of various model parameters. This approach limits the degrees of freedom of the parameter space of the model, simplifying the final calibration of undetermined parameters (see the second part of this study). In addition, new mathematical formulations of important processes have improved the model in terms of the growth of the vector population.

## Background

A warm and humid climate triggers several water-associated diseases, such as malaria [1]. Vector-borne diseases are highly sensitive to global warming and associated changes in precipitation [2]. Malaria is strongly influenced by warm and moist tropical atmospheric conditions [3]. Temperatures in Africa lie above the threshold for parasite development and the rainy seasons lead to a rapid increase of the mosquito population.

At the beginning of the 20th century, Ross was the pioneer who developed the first mathematical model of malaria transmission [4]. Since Ross' work, numerous mathematical malaria models have been developed. One of the most accepted models of malaria transmission dynamics and immunity to date is that of the Garki project [5]. The development of malaria models is hampered when key parameter values are uncertain. For example, so far no general value or satisfying functional relation has been found for the adult mosquito survival probability observed in nature. Most older malaria models further leave out the generation of a variable size of the mosquito population.

Meteorological variables turn out to be useful explanatory variables for the simulation of malaria [6]. Various biological processes depend on temperature, rainfall, and humidity conditions [7]. Climate- or weather-driven malaria models, therefore, allow for a better understanding of the dynamics of malaria transmission. More recently, the construction of dynamic vector models enabled the simulation of a time-dependent mosquito population [8,9].

**Figure 1:**
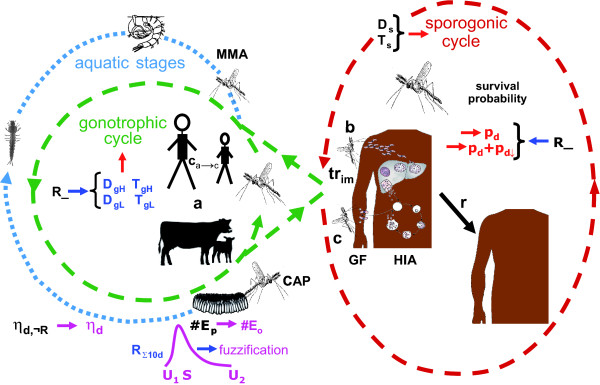
**Components of the LMM**. Illustration of various components of the LMM version of 2010. Blue and red arrows depict the rainfall and temperature dependence of various parts of the model, respectively. The fuzzy logic approach of the oviposition as well as the immature mosquito survival are displayed by pink arrows. Note that abbreviations of model parameters are explained in Table 1.

Hoshen and Morse [10] introduced a weather-driven mathematical biological model of malaria parasite dynamics, known as the *Liverpool Malaria Model *(LMM; see Figure 1 for an illustrative outline of the newly developed LMM version). The LMM comprises weather-dependent within-vector stages as well as weather-independent within-host stages. On a daily basis, the size and behaviour of the total mosquito population and malaria prevalence within human hosts are simulated. The LMM entails the combination of many separate sub-models, each with its own parameterization (see Table 1). The LMM can be used for the understanding of the process of malaria transmission, for mapping purposes, the seasonal forecasting of malaria [11,12], as well as the assessment of the impact of climate change on the disease [13].

**Table 1:** LMM parameters and mathematical formulations

sym	parameter	unit	val_2004_	ref_2004_	val_2010_	ref_2010_	R_lit_
*D_gH_*	humid degree days of the gonotrophic cycle	degree days	37.1	[7]	37.1	[7]	37.1
*D_gL_*	dry degree days of the gonotrophic cycle	degree days	65.4	[7]	65.4	[7]	65.4
*T_gH_*	humid gonotrophic temperature threshold	°C	7.7	[7]	7.7	[7]	7.7
*T_gL_*	dry gonotrophic temperature threshold	°C	4.5	[7]	4.5	[7]	4.5
*R*_	10-day accumulated precipitation threshold	mm	10	NA	10	NA	NA
*R_•_*	rainfall laying multiplier	-	1.0	NA	NU	NA	NA
#*E_p_*	number of produced eggs per female mosquito	eggs	NU	NU	CA	Add. file 1	5-290
#*E_o_*	number of oviposited eggs per female mosquito	eggs	NU	NU	**Eq. 2**	NA	NA
*U*_1_	lower threshold of unsuitable rainfall conditions (fuzzy distribution model)	mm	NU	NU	**0**	[27]	0
*S*	most suitable rainfall condition (fuzzy distribution model)	mm	NU	NU	CA	NA	NA
*U*_2_	upper threshold of unsuitable rainfall conditions (fuzzy distribution model)	mm	NU	NU	CA	NA	NA
*CAP*	cap on the number of fertile mosquitoes	-	10,000	NA	CA	NA	NA
*MMA*	mosquito mature age	days	15	[128]	**12**	Add. files 2 & 3	11.2-30
*η*_*d*,¬*R*_	rainfall independent immature daily mosquito survival probability	%	NU	NU	82.5	Add. file 3	52.7-99.9
*η_d_*	daily immature mosquito survival probability	%	Eq. 3	NA	**Eq. 4**	NA	52.7-89.9
*p_d_*	daily mosquito survival probability	%	Martens I	[59]	**Martens II**	[59]	Add. file 4
*p*_*d*↓_	dry season mosquito survival probability shift	%	NU	NU	CA	NA	Add. file 4
*D_s_*	degree-days of the sporogonic cycle	degree days	111.0	[7]	111.0	[129]	111.0-204.4
*T_s_*	sporogonic temperature threshold	°C	18	[7]	**16**	[7]	14.2-19.0
*a*	human blood index	%	50	NA	**80**	[81,90]	0-100
*b*	mosquito-to-human transmission efficiency	%	50	NA	**30**	Add. file 5	1-50
*c_a→c_*	adult-child conversion rate	-	NU	NU	**0.5**	[125]	0.28-0.5
*HIA*	human infectious age	days	14	NA	**20**	see text	12-30
*r*	daily human recovery rate	day^-1^	0.0284	NA	**0.0050**	e.g., [113]	0.0015-0.0385
*GF*	fraction of gametocyte carriers	%	NU	NU	**50**	Add. file 6	10-70
*c*	human-to-mosquito transmission efficiency	%	50	NA	**20**	Add. file 7	0-37.9
*tr_im_*	trickle of the number of added infectious mosquitoes	-	1.01	NA	1.01	NA	NA

LMM model parameters and mathematical formulations with regard to their original [10] and new settings. Columns: sym: symbol of the model parameter; parameter: name of the parameter; unit: unit; val_2004_: parameter value or mathematical formulation of the LMM_2004_; ref_2004_: LMM_2004 _reference; val_2010_: parameter value or mathematical formulation of the LMM_2010_; ref_2010_: LMM_2010 _reference; R_lit_: literature values. Abbreviations: NU: not used; NA: not available; CA: will be calibrated in the second part of this study [14]. Parameter values and mathematical formulations in **bold **are determined in the present study.

A prerequisite for realistic simulations using malaria models is an optimal set of parameter settings. At the present time, numerous malaria models, including the LMM, are partly based on fairly qualitative assumptions. An optimal set of parameter settings has often not been explored and the models are usually not extensively validated against entomological and parasitological field observations. In the case of the LMM, biological processes were handled somewhat heuristically [10]. The present study aimed to define an optimal parameter setting based on the literature and an improved mathematical formulation of the LMM (this paper), as well as an extensive validation and calibration of the LMM against field studies using quality checked meteorological data as input (second paper [14]). According to Hoshen and Morse [10], the application of the LMM is limited to epidemic malaria areas, as the inclusion of immunity was not part of the model structure. However, it will be shown that the LMM can also be used for endemic malaria areas, where large parts of the population exhibit immunity to infection and disease. The present study illustrates that the refined version of the LMM simulates realistic transmission rates for epidemic as well as endemic malaria areas.

The present study was conducted under the umbrella of the IMPETUS project (*Integrated Approach to the Efficient Management of Scarce Water Resources in West Africa*) [15] and is probably one of the most comprehensive studies to date in terms of gathering knowledge from the literature. Due to the extensiveness of the study, it is split into two parts. In the first part of this study (this paper), changes of the mathematical formulation, which were introduced to better simulate known physical relationships, are described. Further, in this paper the LMM parameter settings are reassessed by means of an extensive literature survey. In the second part [14], meteorological data and malaria observations from West Africa are used for the final calibration of model parameters that lack data from the literature. It will be demonstrated that the calibration by means of field data, results in realistic simulations of disease transmission.

## Methods

### Literature review of entomological and parasitological malaria variables

In terms of malaria modelling, entomological and parasitological data are of particular interest since malaria models have to undergo some form of validation procedure. Information regarding different malaria variables was required for the refinement of the LMM parameter settings. Numerous published malaria observations were extracted from the literature such as from review articles. Data were gathered for the gametocyte prevalence, which is the percentage of humans with gametocytes in their blood. Articles were reviewed in terms of the mosquito survival probability. Horizontal (controlled conditions) and vertical (field conditions) life tables from various studies furnished data for the daily survival probability of aquatic stages of mosquitoes. Furthermore, data were provided by the literature observing the gonotrophic cycle, the number of eggs per female mosquito, the duration of the immature mosquito stages, the duration until asexual and sexual parasites appear in the blood circulation, and the preference towards humans by *Anopheles *females. In addition, values for the transmission efficiencies of the malaria parasite between the human and mosquito hosts were searched for in the literature.

### Changes of the set of parameter settings

The LMM consists of a number of parameters that have to be set for the simulation of the malaria transmission cycle. In the first part of this study, the parameter settings and mathematical formulation of the LMM is reviewed and refined according to the gathered literature data. Sub-modules of the LMM are modified in order to handle specific model problems. For example, in the current version of the model, too many infective mosquito bites are generated in humid areas such as the equatorial tropics (see Figure Six in [14]). In order to refine the model parameter settings and mathematical formulations, the following strategy was applied: (i) Model parameters lacking numerous literature references such as those related to the gonotrophic or sporogonic cycle were not changed. (ii) Parameters exhibiting various values in the literature were reassessed if possible (bold values and formulations in Table 1). As a result of an extensive literature survey, entomological and parasitological malaria data were gathered. (iii) In the second part of this study [14], uncertain model parameters and those parameters that lack literature references are calibrated.

The strategy was to set as many parameters as possible, in order to simplify the final calibration of the model by means of entomological and parasitological observations from West Africa. The selected procedure significantly reduces the degrees of freedom of the parameter space, and the literature survey ensures a refined setting of individual model parameters.

LMM simulations reveal that various model parameters exert the same effects on the model behaviour [13]. In terms of malaria transmission, for example, the *human blood index *(*a*) can be compensated by a lower value of the *number of produced eggs per female mosquito *(*#E_p_*). For this reason, the final calibration (see [14]) will compensate some uncertain assessments of predefined model parameters.

### Changes of LMM modules

The simulation of some key processes is changed in the new *LMM version of 2010 *(henceforth called LMM_2010_) to achieve a higher biological and physical accuracy. For example, the process of oviposition, the immature survival probability, as well as the mosquito survival schemes are reviewed. In the original *LMM version of 2004 *(henceforth called LMM_2004_), some detailed aspects of parasitological processes as well as age-dependencies of the malaria disease in humans are not included. Some of these aspects are newly introduced in the model.

## Results

The LMM simulates the spread of malaria at a daily time resolution, using* daily mean temperature *(*T*) and *10-day accumulated precipitation *(*R*_Σ10*d*_). For a thorough mathematical formulation of the LMM_2004 _version the reader is referred to Hoshen and Morse [10]. In this paper, the mathematical formulations of important processes are refined, and parameters of the LMM are reviewed and changed if necessary.

### Review of the LMM set of parameter settings and mathematical formulation

#### Oviposition

A realistic simulation of the size of the mosquito population is a prerequisite for the simulation of malaria transmission in the human population [8,16-18]. Ovipositioning is dependent on open water bodies that are mostly created by precipitation events. In the LMM_2004 _version, the number of laid eggs is roughly assumed to be proportional to both the number of ovipositing mosquitoes and to the 10-day accumulated rainfall (*R*_Σ10*d*_). The constant of proportionality is the so-called *rainfall laying multiplier *(*R_•_*), which couples *R*_Σ10*d *_with the number of ovipositioning female mosquitoes.

Observations show that the *number of produced eggs per female mosquito *(#*E_p_*; i.e. the number of eggs/mature oocytes that are found by dissection and/or the oviposition of females) depends on the body size of female mosquitoes [19] as well as on the age of the females [20]. As shown (see Additional file 1) #*E_p _*ranges between 5 and 290 eggs [19-21]. For this reason, the LMM_2010 _version takes into account a realistic number of eggs per *Anopheles *female (i.e. accounted by #*E_p _*and is determined in the second part of this study [14]). However, due to environmental conditions, not all produced eggs are (successfully) oviposited [22,23].

In the LMM_2010_, in terms of the deposition of eggs as well as the immature survival a fuzzy logic approach is used: The availability of suitable mosquito habitats is not a simple linear function of rainfall [24]. Certain rainfall regimes will be more suitable, and probably no further breeding sites are provided with increasing rainfall amounts. Various studies have noted that breeding places are washed out by strong rainfall events [24-26]. In fact, rainfall significantly affects larvae by flushing them out of their aquatic habitat and killing them [23]. For these reasons, the LMM_2010 _uses a simple fuzzy distribution model comparable to Craig *et al. *[27], which applies *R*_Σ10*d *_as input. The general concept is the following: (i) none or a small amount of eggs are oviposited during dry conditions; (ii) more moist conditions lead to a higher proportion of deposited eggs; and (iii) breeding places are washed out by excessive rainfall. The fuzzy logic approach, therefore, differentiates between *dry unsuitable conditions *(threshold *U*_1_), a *most suitable condition *(*S*), and again *unsuitable conditions due to very high rainfall *(threshold *U*_2_). Obviously, *U*_1 _is set to zero since female mosquitoes are not able to produce progeny without water supply. This fuzzy distribution model might reflect a more physically correct relationship of the egg laying process than the construction with *R_• _*used in the LMM_2004_.

**Figure 2:**
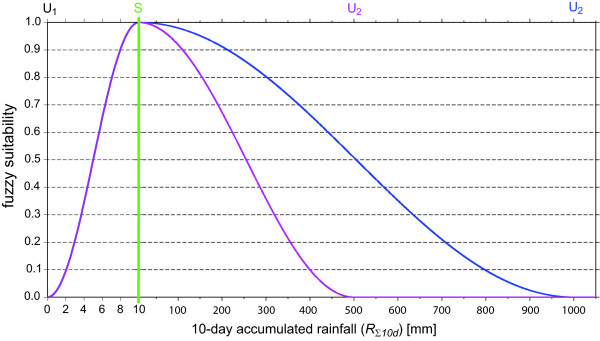
**Fuzzy distribution model**. Illustration of the fuzzy function with regard to the influence of the 10-day accumulated rainfall (*R*_Σ10*d*_) on the number of oviposited eggs per female mosquito (#*E_o_*) as well as the daily immature mosquito survival probability (*η_d_*). The green vertical line at 10 mm (= *S*) depicts the most suitable rainfall conditions and separates different scales of the abscissa. Pink and blue lines depict two different settings of the fuzzy distribution model. According to these adjustments rainfall condition are unsuitable for *R*_Σ10*d *_values of 0 mm (= *U*_1_) and above of 500 or 1000 mm (= *U*_2_), respectively.

The fuzzy distribution model computes fractions between zero (conditions unsuitable, *U*_1 _and *U*_2_) and one (condition most suitable, *S*). The *fuzzy suitability *(*f*) of *R*_Σ10*d *_is computed by means of a sigmoidal fuzzy membership curve (see also Figure 2):

(1) $$f\left(R_{\Sigma 10d}\right)=\{\begin{array}{ll} 1-cos^{2}\left(\frac{R_{\Sigma 10d}-U_{1}}{S-U_{1}}\frac{\pi }{2}\right), & \text{if}\;U_{1}<R_{\Sigma 10d}<S\\ \;cos^{2}\left(\frac{R_{\Sigma 10d}-S}{U_{2}-S}\frac{\pi }{2}\right), & \text{if}\;S<R_{\Sigma 10d}<U_{2}\\ \;\;\;\;0, & \text{else} \end{array}$$

The final *number of oviposited eggs per female mosquito *(*#E_o_*), which forms the basis of the modelled immature mosquito population, is simply determined by the multiplication of the #*E_p _*with the respective value of the fuzzy function (Eq. 1), that is:

(2) $$\#E_{o}=\#E_{p}\cdot f\left(R_{\Sigma 10d}\right)$$

Due to the protective effect of houses or the usage of mosquito nets, only a limited number of mosquitoes are able to come into contact with humans. Humans, therefore, can only be exposed to a certain number of biting mosquitoes. Environmental conditions also have an impact on the growth of the mosquito population. Due to the limited flight range of mosquitoes of less than seven kilometres [28] only a limited number of breeding sites are available for *Anopheles *females. Provided that there are a large number of fertile mosquitoes, the larval densities will increase under such circumstances and will hence produce higher larval mortalities [29-32]. Takken *et al. *[19] showed that high larval densities lead to a higher mortality and a slower gonotrophic development of adult mosquitoes due to reduced body sizes and, therefore, small nutritional resources.

All the environmental and physical causes, outlined above, are combined in another model parameter limiting the number of fertile mosquitoes. The *cap on the number of fertile mosquitoes *(*CAP*) simply restricts the size of the mosquito population to a certain level. Without the application of *CAP *the growth of the mosquito population is often unrealistically large. Note that the size of the number of fertile mosquitoes is also limited in the LMM_2004_, where *CAP *has been set to 10,000. However, *CAP *will be calibrated to a much lower value in the LMM_2010 _in the second part of this study [14].

#### Mosquito Mature Age (MMA)

Immature mosquitoes undergo the egg, larval, and pupal stages until they mature to adult mosquitoes. In the LMM_2004_, the *Mosquito Mature Age *(*MMA*; i.e. the time between the egg stage and adult emergence) is fixed at 15 days. However, field studies in Kenya and Mali showed (see Additional file 2) that on average the time between oviposition and eclosion is about 12 days [33-37]. On this account *MMA *is reduced from 15 to 12 days in the LMM_2010_.

#### Survival of immature mosquitoes

The life cycle of mosquitoes comprises the egg, larval, pupal, and adult stages. The egg, larval, and pupal stages are entirely aquatic and, therefore, mostly depend on weather conditions. Besides climatic conditions, competition due to overcrowding, water quality, food supply, cannibalism, predators, parasites, as well as pathogens are limiting factors for aquatic stages of mosquitoes [23,32,34,38,39]. In the LMM_2004_, the *daily survival probability of immature mosquitoes *(*η_d _*) is only subject to *R*_Σ10*d *_and is calculated as follows:

(3) $$\eta_{d}=\frac{1+R_{\Sigma 10d}}{2+R_{\Sigma 10d}}$$

Therefore, also under small precipitation amounts, a large fraction of larvae outlives the maturation period of 15 days (i.e. the mature age of mosquitoes of the LMM_2004_). For example, 27.1 and 49.8% of the larvae become adults in the model at a constant *R*_Σ10*d *_value of 10 and 20 mm, respectively. However, age distributions from so-called vertical life tables from field studies (see Additional file 2) reveal that a much smaller fraction (2-15%) of deposited eggs emerge to adults [33-35,37,40,41]. By contrast, most laboratory studies prove by means of so-called horizontal life tables (see Additional file 3) that under controlled conditions more than 90% of eggs, larvae, and pupae survive one day [29-32,36]. The higher laboratory survival is because under controlled conditions various natural factors are eliminated.

In the LMM_2010_, the calculation of the survival of immature mosquitoes is separated into two parts. In a first step, it is assumed that survival is independent of hydrological conditions. The *rainfall-independent daily survival probability of immature mosquitoes *(*η*_*d*,¬*R*_) is set to 82.5%. This is due to the fact that in general less than 10% of the immature mosquitoes reach the adult stage under field conditions (see Additional file 2) and because the *MMA *is fixed to twelve days (0.825^12 ^≈ 0.099). In a second step, the dependence on the hydrological stage is included. For simplicity, the same parameters are used as for the fuzzy logic approach of the oviposition (*U*_1_, *S*, and *U*_2_; Table 1). The survival probability of immature mosquitoes is realized by the multiplication of *η*_*d*,¬R _with the fuzzy value:

(4) $$\eta_{d}=\eta_{d,\neg R}\cdot f\left(R_{\Sigma 10d}\right)$$

As a consequence, in the LMM_2010 _the *η_d _*can reach at maximum 82.5% and no more than 10% of the oviposited eggs emerge to adults (i.e. about 5.4% and 0.7% for *f *= 0.95 and *f *= 0.8, respectively; f: value of the fuzzy distribution model). This modelling approach again reflects a more physically correct relationship than the original *η_d _*equation (see Eq. 3). Note that Pascual *et al. *[9] used a comparable approach in their model. Larval mortality was simulated as a function of accumulated days with no rain to represent desiccation of breeding sites. What is not considered is the fact that mosquito larvae can benefit from drought conditions such as when streams dry up due the occurrence of numerous pools [42-44]. Also the existence of permanent breeding sites provided by, for example, large ponds, lakes, and rivers is neglected in both versions of the model.

#### Survival probability of adult mosquitoes (p_d_)

The age structure of *Anopheles *females and survival rate exerts a strong influence on the reproduction rate of the mosquito population and the spread of the malaria parasite. Hence, the vector survivorship is of paramount ecological importance for the distribution of malaria [45-49]. The *daily survival probability of female mosquitoes *(*p_d_*) depends on characteristics of mosquito species, activities of individuals, climatic conditions, the incidence of parasites, predators [50], and the mosquito age [51]. Most of these factors are elusive to observe and are only indirectly taken into account in malaria models. The LMM only considers the weather impact on the vector survivorship. However, there is only limited information available from entomological field campaigns in terms of the dependence of the adult mosquito survival on temperatures. With regard to climate the survival is affected by temperature and the relative humidity [52,53]. At daily mean temperatures of about 5°C or even lower malaria vectors seem to disappear [27]. The entomological study of Kirby and Lindsay [54] clearly showed that extremely high temperatures above 40°C are often fatal to mosquitoes. Note that for simplicity the LMM uses an exponential model of mortality. Most observed *p_d _*values range between about 80 and 95% (see Additional file 4).

**Figure 3:**
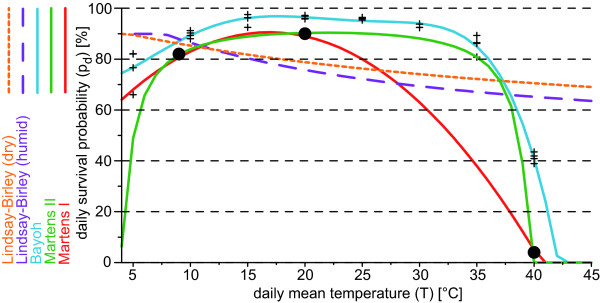
**Mosquito survival schemes**. Illustration of different schemes regarding the daily mosquito survival (*p_d_*) against the daily mean temperature (*T*): the Lindsay-Birley (humid (dry) conditions in dashed purple (orange)), the Martens I (red line; derived from [57-59]), the Martens II (green line; given by [27] and [59]), and the Bayoh scheme (blue line; derived from [61]). Crosses (+) denote *p_d _*values with regard to different temperature and humidity conditions (see text). In addition, the data basis of the two Martens schemes is inserted as dots (*•*).

Various daily mosquito survival probability schemes (*p_d_*-scheme) were developed with regard to malaria modelling. In the LMM, four different *p_d_*-schemes are implemented, these are: the so-called *Lindsay-Birley*, the *Martens I*, the *Martens II*, and the *Bayoh scheme *(Figure 3). Initially, the LMM was set by the Lindsay-Birley scheme [10,55]. However, it is not clear whether vector survival per gonotrophic cycle is constant [10]. This *p_d_*-scheme is furthermore unrealistic at very high temperatures. Experiments performed by Kirby and Lindsay [54] showed that 50% of *Anopheles arabiensis *and *Anopheles gambiae s.s*. are killed at 40°C within at least two hours. In contrast, above 40°C the Lindsay-Birley scheme shows unrealistic high survivorships (cf. Figure 3).

The literature [27,56] refers to studies published by Martens [57-59]. Martens [59] states (see also [57,58]): Relying on data reported by Boyd [50], Horsfall [60], and Clements and Paterson [46], a daily survival probability of 0.82, 0.90, and 0.04 at temperatures of 9, 20, and 40°C is assumed, respectively, expressed as:

(5) $$p_{d}=exp(\frac{-1}{-4.4+1.31\;T-0.03\;T^{2}}).$$

The so-called Martens I scheme was obviously generated as a polynomial connecting the quoted three data points in the *T*-*p_d_*-diagram (Figure 3) and is based on the following equation:

(6) $$p_{d}=-0.0016T^{2}+0.054T+0.45$$

The formula (Eq. 5) provided by Martens [59] is not used in the LMM_2004_. However, in the LMM_2010 _this formula is introduced and forms the so-called Martens II scheme. The main difference between the Martens I and II schemes is the earlier and smoother decrease of *p_d _*at temperatures above 25°C in the Martens I scheme.

Taking into account the uncertainty of the so far introduced *p_d _*-schemes, further data are needed. Bayoh [61] observed the survival and mortality rates of *Anopheles gambiae s.s*. in environmental chambers at combinations of temperatures from 0-45°C at 5°C intervals and relative humidities of 40%, 60%, 80%, and 100%. Using the data from these experiments and assuming an exponential model of mortality it is possible to derive *p_d _*values. The identified probabilities did not vary considerably with regard to different humidities. For this reason, the probabilities were averaged at each temperature. Finally, the average was used to define a polynomial regarding *Anopheles gambiae s.s*. survivorship in the laboratory, the so-called Bayoh scheme (Figure 3):

(7) pd=−2.123 10−7 T5+1.951 10−5 T4−6.394 10−4 T3 +8.217 10−3 T2−1.865 10−2 T+7.238 10−1

As previously discussed, vector survival is higher in captivity than in the wild and hence *p_d _*is generally higher in the Bayoh scheme than in both Martens schemes. It is interesting to note that the Bayoh scheme reveals only a slight decrease of *p_d _*between 25 and 35°C. For this reason, the Bayoh scheme agrees better with the Martens II than with the Martens I scheme. On account of these facts the Martens II scheme is utilized for the LMM_2010 _version.

Various studies point out the importance of the atmospheric humidity on the longevity of adult vectors [58,62-66]. Relative humidities above 60% seem to be preferred by most vector species. However, it is noted that the crucial factor for the physiology of *Anopheles *females might be the absolute saturation deficit rather than the relative humidity [67]. The usual dryness of the atmosphere in arid or semi-arid areas such as the Sahel militates against the longevity of mosquitoes and thus reducing malaria transmission [68]. In Niger, for example, *Anopheles *populations seem to drop steeply around October, when shifts in the prevailing winds drastically reduce humidity. Favourable microclimates become gradually scarcer as the Harmattan conditions establish and the dry season progresses (A. Kiszewsky, personal communication, 2006). In El Salvador, Weidhaas *et al. *[40] calculated lower adult survival rates for *Anopheles albimanus *during the dry than during the rainy season. Daily survival was 65-70% and 73-91%, respectively. However, the authors note that the occurrence of breeding outside the study area and the immigration of mosquitoes might be in part responsible for the higher calculated rainy season survival. On account of the possible influence of humidity on vector survival a *shift of the dry season mosquito survival probability *(*p*_*d*↓_) is introduced in the LMM_2010 _version and is set in the second part of this study [14]. To simplify matters, *p*_*d*↓ _is applied when the 10-day rainfall amount(*R*_Σ10*d*_) is lower than the *10-day accumulated precipitation threshold *(*R*_) that distinguishes between dry and humid weather conditions [10].

#### Sporogonic cycle

The sporogonic cycle or extrinsic incubation denotes the development of the malaria parasite within the mosquito vector. The development of sporozoites is temperature dependent [7,69]. There is an uncertainty about the value of the *sporogonic temperature threshold *(*T_s_*; note that *T_s _*refers to the daily average temperature). Lindsay and Birley [63] concluded that the parasite development ceases below temperatures between 14.5 and 16°C for *Plasmodium vivax *and between 16 and 19°C for *Plasmodium falciparum*. It is not surprising that the *T_s _*data given in the literature are inconsistent. Various publications agree that the *T_s _*is located within a certain range [57,58,62,63,70]. On the other hand a temperature threshold of 18°C is referred to in various publications [1,10,71-73], whereas others quote a value of 16°C or even lower [27,43,56,59,65,74-81].

The setting of the threshold is particularly important when malaria is modelled in areas with temperatures in the range of *T_s _*(e.g., in highlands of East Africa). For temperatures well above *T_s _*the length of the sporogonic cycle is much less dependent upon the setting of the lower threshold temperature. Regarding the sporogonic cycle the LMM_2004 _was set at a threshold of 18°C [10]. However, modelled temperatures or data from weather stations are unlikely to record conditions in the microhabitats where vectors spend most of their time [53,82]. For example, indoor temperatures in the Usambara mountains (northeast Tanzania) have found to be 2.6°C higher than outdoor air temperatures [83,84]. By resting in more climatically stable and warmer houses, vectors may avoid cold temperatures and thus the restrictions concerning the progress of the parasite development [64,70,85]. Therefore, the effect of altitude might be partly compensated when mosquitoes stay in heated houses [86]. For this reason, the use of 16°C as a temperature threshold for parasite development is used for the LMM_2010_.

#### Human blood index (a)

The rate of malaria transmission directly depends on the degree of the host-vector-pathogen contact. *Anopheles *mosquitoes with a high preference for human blood are considered important vectors of malaria [87]. This fact is expressed in the so-called *human blood index *(*a*) that is the proportion of blood meals of a mosquito population obtained from humans rather than animals, for example, cattle.

The assessment of *a *is a difficult task as it is dependent on the feeding preference of each species, the accessibility of different potential hosts, as well as on the mosquito sampling technique. The calculation of *a *is most often performed by captures of indoor resting mosquitoes (endophilic females) excluding exophilic mosquitoes feeding on humans [88]. By contrast, *a *is best estimated by applying the unweighted mean of a part-sample collected from human dwellings and one from other types of resting-place [89]. Kiszewski *et al. *[81] and Moffet *et al. *[90] presented median and mean values of *a *from four and ten African *Anopheles *vectors, respectively. The major malaria vectors in Africa *Anopheles arabiensis*, *Anopheles gambiae s.s*., and *Anopheles funestus *show fairly high values of *a*. Except for the mean value of *a *of *Anopheles arabiensis *all the median and mean values of these vectors are consistently higher than 80% for these three vectors. For this reason, the LMM_2004 _value of *a *of 50% seems to be an underestimation. Due to the fact that the endophily of major African vectors probably was overestimated [88,91] the value for LMM_2010 _is approximated by 80%.

#### Mosquito-to-human transmission efficiency (b)

Not every biting infectious mosquito is able to pass malaria infection by injecting parasites into humans. Unfortunately, the *mosquito-to-human transmission efficiency *(*b*; i.e. the proportion of sporozoite-positive mosquito bites infecting susceptible people; see Additional file 5) is a largely undefined parameter [92]. For this reason, this factor is commonly ignored in most malaria models [93]. However, the proportion of actually infective *Anophelines *is a crucial parameter in the epidemiology and simulation of malaria.

One infectious bite is generally thought to infect about half of immunologically-naive people and this level seems to decrease with the level of endemicity and is age dependent [94]. This transmission efficiency is a function of the exposure history, reflecting effects of immunity [95]. The study of Rickman *et al. *[96] showed that three (two) out of five non-immune subjects developed malaria parasitaemia after the exposure to one (two) infected mosquito(es) (that means *b *= 33%). In addition, a total of 44.1% of 68 experimentally infected *Anopheles gambiae *and 49.2% of 63 infectious *Anopheles stephensi *transmitted sporozoites in vitro into a sucrose solution [97]. By contrast, surveys of infants revealed fairly low *b *values. For example, Pull and Grab [98] estimated the value of *b *as between 1.5 and 2.6%. Indeed, such studies generally ignore superinfection and the fact that adults are bitten more often than children or infants [99]. Superinfection also explains the strong variation of *b *in children in an urban area of Senegal, where age-corrected *Human Biting Rate *(*HBR*) values were used for the analysis of *b *[91]. The low observed *Entomological Inoculation Rate *(*EIR*) values in March led to comparatively high computed *b *values of about 46%. By contrast, the stronger transmission in June resulted in the calculation of *b *of only about 8% [91]. In this context it should be noted that there is no reason for the seasonal variation of *b*. In conclusion, the value of *b *seems to be generally lower than 50% - the value of the LMM_2004 _- for most African populations. For this reason, *b *is approximated as 30% in the LMM_2010 _version.

#### Human Infectious Age (HIA)

The transmission of the malaria parasite from humans to mosquitoes is made possible by male and female gametocytes. The duration after infection until mature gametocytes appear in the blood is termed here as the *Human Infectious Age *(*HIA*). The duration in days after that a human becomes infectious starting from the mosquito bite is longer than the so-called *prepatent period *(*n_p_*; i.e. the time needed for the detection of asexual parasites in the blood after the infection of humans). This is due to the *time needed for gametocytogenesis *(*n_mf_*; i.e. the time needed for the production of male and female gametocytes), which is also called sequestration time, as well as the final *maturation period of gametocytes *(*n_m_*). Therefore, *HIA *is computed via *HIA *= *n_p _*+ *n_mf _*+ *n_m_*.

Asexual parasites are usually detected by blood slides, which are examined under a microscope. According to microscope detection *n_p _*lasts one week or slightly longer (e.g., [100]: eight days). Schneider *et al. *[101] compared the microscope with the *QuanTitative-Nucleic Acid Sequence-Based Amplification *(QT-NASBA) detection method. They found that the microscope detection is delayed by one to two days (*n_p_*: 8.3 versus 6.0-7.0 days). This is in agreement with the findings of Murphy *et al. *[102], who cultured asexual parasites from blood taken 6.5-7.0 days after exposure. By contrast, Rickman *et al. *[96] found a prolonged *n_p _*of 14.0-16.5 days from patients without antimalarial immunity. Moreover, a study comparing the Panama, McLendon, and Santee Cooper strain of *Plasmodium falciparum *revealed mean *n_p _*values of 10.3, 13.0 and 9.8 days, respectively [103].

The duration for gametocytogenesis (*n_mf_*) is derived in vitro or from the delay in vivo between the onset of symptoms (e.g., fever) or the detection of asexual parasites and the detection of male and female gametocytes [104]. The values reported in the literature generally range between 7 and 15 days ([105]: about ten days for non-immune subjects; [106]: about twelve days for immune adults; [107]: nine to twelve days; [108]: 7-15 days). Diebner *et al. *[109] and Eichner *et al. *[104] more recently estimated the sequestration time from fitting a model to malaria therapy data. According to their studies the time needed for the transition of asexual blood stages of *Plasmodium falciparum *to mature gametocytes amounts to four to twelve days (mean 7.4 days). It is also shown that sequestration time depends on presence of the parasite strain (geometric mean: 4.9 days for Santee Cooper strain (South Carolina, 1946); 6.2 days for El Limon strain (Panama, 1948); 8.7 days for McLendon strain (South Carolina, 1940)). Eichner *et al. *[104] concluded that in the former literature the time for sequestration was probably overestimated by the time needed to reach a certain level of gametocytaemia that can be detected by microscopy. However, gametocytes of *Plasmodium falciparum *do not infect mosquitoes when the mature forms first appear in the blood. The time needed for *n_m _*is about one to four days, when these forms of the malaria parasite finally become capacitated [68,110,111].

In summary, due to the length of *n_p _*(about six to ten days), *n_mf _*(four to twelve days), and *n_m _*(one to four days) *HIA *lasts altogether about 11-26 days. For this reason, *HIA *is approximated as 20 days in the LMM_2010_, which is five days longer than the LMM_2004 _value of 15 days (cf. Table 1).

#### Recovery rate (r)

A low *recovery rate *(*r*) of malaria infection is a crucial factor for transmission of malaria. Recovery is affected by the genetic multiplicity of the malaria parasite and is a function of the exposure history, reflecting effects of immunity. Parasite clearance is, therefore, closely related to the age of an individual as well as to the transmission intensity. The former fact was found in data from longitudinal studies from 16 villages in the West African savannah [112]. Daily recovery rates were 0.0045 in infants (< 1 year), fell to a minimum of 0.0016 in young children (1-4 years), and increased again to 0.0194 in the oldest adult age group (≥43 years). The dependence of *r *on the transmission intensity was found at 30 sites along coastal Kenya. Gu *et al. *[95] showed that the daily parasite clearance was lower than 0.005 day^-1 ^at one or less infectious bites per year and higher at intensities of ten or more.

The mathematical formulation of the LMM does not account for an individual immune status. As a result, *r *is independent from transmission intensity or age of an individual in the model, only one single setting of the recovery rate is possible. For this reason, the applied clearance rate represents an age or transmission intensity average. Due to the fact that the LMM_2004 _does not include superinfection, parasite clearance is related to the elimination of single parasite clones [10]. The recovery rate was originally set to 0.0284 day^-1 ^enabling about 90% of the infected population to clear their infection after 80 days ((1 - *r*)^80 ^≈ 0.10). However, estimates from simple infections of *Plasmodium falciparum *induced in immunologically naive patients for malaria therapy often revealed longer persistence. Patterns of recrudescence survived partly longer than 150 days [100]. In order to partly take into account superinfection, the parasite clearance is significantly decreased in the LMM_2010 _and is set to 0.005 day^-1^. In fact, the value of 0.005 day^-1 ^was previously assessed by Macdonald and Gockel [113] and was applied in various malaria models [95,114]. Note that the reduction of the *r *value is essential when the LMM is extended to endemic malaria areas.

#### Gametocyte prevalence (sPR)

The presence of male and female gametocytes in the blood of a human host, the so-called *sexual Parasite Ratio *(*sPR*; i.e. gametocytaemia), is a necessary condition for malaria transmission. Gametocytaemia is generally lower than the *parasite ratio *(*PR*). Only one annual mean *sPR *value was found to be higher than 40%, which has been detected by the *Reverse Transcriptase-Polymerase Chain Reaction *(RT-PCR) (see Additional file 6). In most studies using microscopy less than 15% of the population were detected as gametocyte carriers. In contrast, the majority of studies revealed asexual parasite ratios above 30%.

There is a problem of enumerating gametocytes patterns. Gametocytes are prone to be missed by standard microscopy examination [111,115]. For example, RT-PCR revealed in comparison with microscopy a 40% higher *sPR *[116]. Ouedraogo *et al. *[117] recently found that the QT-NASBA technique provided about 3.3 fold higher estimates of *sPR *than microscopy. This clearly demonstrates that studies based on the detection of gametocytes by microscopy are rather insensitive and inaccurate in the quantification of gametocytes in blood smears.

Sexual and asexual parasite ratios are generally higher in children than in adults. That is due to the fact that adults better control asexual and sexual parasite densities, and are, therefore, more likely to carry gametocytes at the borderline level of detection [115]. Young children are unlikely to be able to control malaria infections, and likely more parasites turn into gametocytes. In Kenya, Bousema *et al. *[118] found a decrease in the mean duration of gametocyte carriage with increasing age for asymptomatic children.

The fact that not all infected humans actually carry male and female gametocytes is accounted for in the LMM_2010 _version. Note that this detailed aspect is not included in the LMM_2004_. A *fraction of gametocyte carriers *(*GF*) is introduced into the model. This fraction stands for the proportion of the population that: (i) is infected by the malaria parasite; (ii) has already passed the human infectious age (*HIA*); and (iii) is exhibiting a sufficiently high density of gametocytes. These humans are, therefore, the infectors of the human population. Due to the problem of enumerating gametocytes patterns, the GF is set to the comparatively high value of 0.5 in the LMM_2010_.

#### Human-to-mosquito transmission efficiency (c)

Not all *Anopheles *females feeding on gametocyte-infected hosts get infected. Most malaria transmission models have not used direct field estimates of the *human-to-mosquito transmission efficiency *(*c*; i.e. the proportion of mosquito bites on infectious humans, which infect susceptible mosquitoes) that is usually termed parameter *c *in the literature [119].

One factor reducing the infectivity of gametocytes to mosquitoes is transmission-blocking immunity: a specific immunity acquired in humans. Immune factors, ingested with the blood meal, inhibit or block the development of the free sexual stages: gamete, zygote, and ookinete, which have common antigens with gametocytes [120].

The infectiousness of mosquitoes can be determined by using blood from gametocyte carriers. It is either measured by direct skin feeding or by membrane feeding [121]. However, the best method for estimating infectiousness of a human population is to feed laboratory-reared *Anopheles *on a representative population sample without regard to the presence of gametocytes [122]. Obviously, not all *Anophelines *feeding on gametocyte-infected hosts become infected. Human-to-mosquito transmission efficiencies are generally lower than 40%, and for the majority of trials infectiousness is higher than 20% (see Additional file 7). Muirhead-Thomson [123] observed that the 'best infectors' infected only about 30% of mosquitoes feeding on them. On the other hand, cryptic gametocytaemia can result in mosquito infections [124]. In the LMM_2010 _version, *c *is approximated as 20% in contrast to 50% of the LMM_2004_, which is located amidst the observed measurements. This means, in combination with the value of *GF*, that a fraction of 10% of females feeding on gametocyte-infected hosts becomes infected with the parasite in the model.

#### Issues regarding the age-dependence of malaria

Entomological and parasitological studies clearly identified the age-dependence of malaria in areas of year-round and seasonal malaria transmission. The increase of functional immunity from child- to adulthood leads to an age-dependence of various malaria parameters (these are: *PR*, *sPR*, *r*, *b*, as well as *c*). The values differ considerably between children than in adults.

Some individuals are more likely to be bitten than others. Port *et al. *[99] found that the proportions of feeds upon an individual human are associated with the body surface of the host. Their results from The Gambia revealed a child-to-adult conversion factor of 3.57. Such a heterogeneous biting pattern was also found in a village close to Brazzaville (Congo). Number of bites increased regularly in infants (age: <2 years), children (2-10 years), adolescents (10-20 years), and adults, in proportions of 1:1.93:2.53:3.00 [125] implying a child-to-adult conversion factor of 1.43. Note that averaged proportions of adolescents and adults were used (1.93·1.43 ≈ 2.765 = $\frac{2.53+3.00}{2}$)

The parameter settings in the present study refer to observations from children, since data values (e.g., that of *PR*) are mostly available for this population group. This in turn implies that the model output (e.g., *PR*, *HBR*, *EIR*) is again related to that of children. Due to the lack of an age-dependence of the LMM it is assumed that *children between 2-10 years *(*groupC*) and the *rest of the population *(*groupR*; these are infants, adolescents, and adults) equally contribute to the infectious reservoir of malaria. On that condition an isolated simulation of the malaria transmission based on *groupC*, *groupR*, or that of the whole population always results in the same infection level of the mosquito population and after age-adjustment also in the same *HBR *and *EIR *values. The LMM simulation is, therefore, henceforth orientated on *groupC*. For this reason, the host-vector contact is lowered in the LMM_2010 _version by the *adult-child conversion rate *(*c*_*a*→*c*_). Due to findings of Port *et al. *[99] and Carnevale *et al. *[125]*c_a→c _*is approximated as 0.5, which means that *HBR *and *EIR *values for children between 2-10 years are about two times lower than that for adults. This in turn implies that simulated *HBR *and *EIR *values must be doubled when they are compared to field observations.

## Discussion

**Figure 4:**
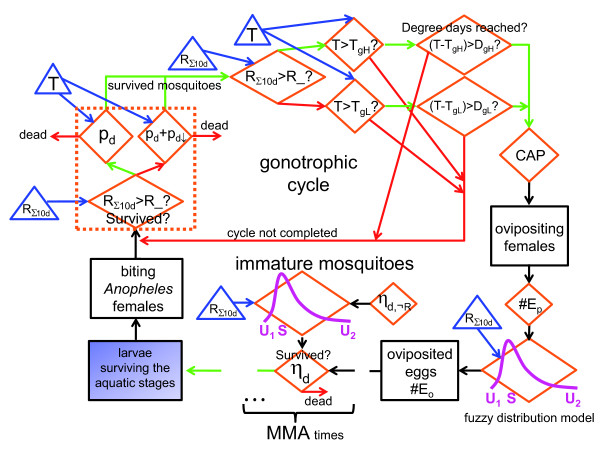
**Flow chart of the simulation of the mosquito population**. Flow chart of various components of the LMM version of 2010 regarding the simulation of the mosquito population. The gonotrophic cycle as well as the development of immature mosquitoes within the aquatic stages are illustrated. Individual states of immature and mature mosquitoes are indicated by black rectangles. The orange rhombi denote decisions within the model as well as implemented functions. Green and red arrows represent a positive and negative affirmation, respectively. The impact of the model drivers is indicated via blue triangles and blue arrows (*T*: daily mean temperature; *R*_Σ10*d*_: 10-day accumulated rainfall). Note that abbreviations of model parameters are explained in Table 1.

**Figure 5:**
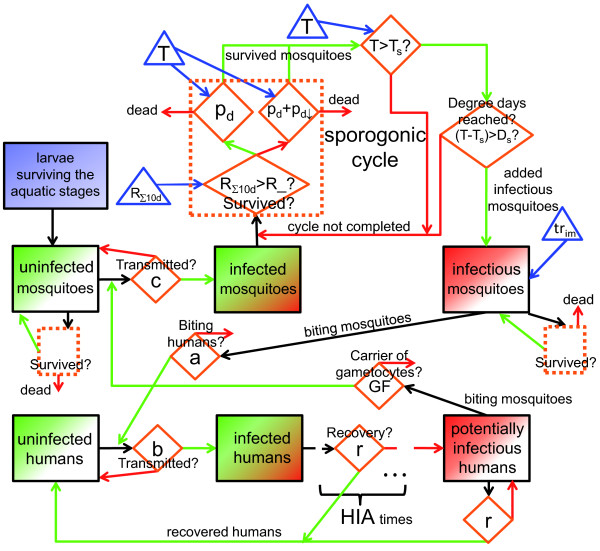
**Flow chart of the malaria parasite transmission between humans and mosquitoes**. Flow chart of various components of the LMM version of 2010 in terms of the modelling of the malaria parasite transmission between the human and mosquito populations. The sporogonic cycle of infected female mosquitoes is furthermore displayed. Individual states of humans and mosquitoes are indicated by black rectangles. The orange rhombi denote decisions within the model as well as implemented functions. Green and red arrows represent a positive and negative affirmation, respectively. The impact of model drivers is indicated via blue triangles and blue arrows (*T*: daily mean temperature; *R*_Σ10*d*_: 10-day accumulated rainfall; *tr_im_*: trickle of the number of added infectious mosquitoes). Note that abbreviations of model parameters are explained in Table 1.

The aim of the first part of the present study was the development of a refined parameter setting and mathematical formulation of the LMM (cf. the various components of the LMM_2010 _outlined by flow charts in Figures 4 and 5). For this reason, key model parameters as well as some modules of the original LMM were reviewed. It was found that the knowledge gathered by entomological and parasitological field research enabled the final setting of most model parameters. Various parameters were, therefore, reassessed by means of an extensive literature survey. The setting of numerous model parameters furthermore reduces the degrees of freedom of the model, which simplifies the final calibration of the model in the second part of this study [14]. In addition, important malaria processes such as the vector survival during aquatic stages were changed in the model to achieve a higher biological and physical accuracy (Figure 4).

Most model parameters were set based on data and knowledge currently available in the literature. However, various model parameters lack a precise setting due to variable observed values, which are probably a result of different environmental conditions. As in other malaria models (e.g, [8,18]), various model parameters were set by a consensus literature value and a parameter range is indicated for most parameters (see Table 1). Comparable with previous studies (e.g., [5,18]), the remaining undetermined parameters will be calibrated by means of field observations [14]. As mentioned earlier, the final calibration (see [14]) will largely compensate potential inaccurate assessments of the predefined model parameters. Some newly inserted (these are *S*, *U*_2_, #*E_p_*, and *p*_*d*↓_; see Table 1 for their explanation) and one old parameter (i.e. *CAP*) of the LMM_2010 _were not set due to the lack of (precise) information in the literature. In the second part of this study [14], such parameters are calibrated by means of entomological and parasitological data from West Africa.

One of the most important aspects of malaria transmission is the size of the mosquito population, which strongly depends upon breeding conditions. The relationship between rainfall and larval breeding was treated in a very simple approach in the original version of the LMM. In the current model version, ovipositioning and immature mosquito survival is controlled by the fuzzy distribution model (Figure 4). As will be shown in the second part of this study [14] the fuzzy logic approach seems to be more realistic than the former linear rainfall relationship with regard to the oviposition. In addition, the survival through the aquatic stages was adjusted to the field conditions. In contrast to the LMM_2004_, the fuzzy distribution model ensures that only a reasonable fraction of oviposited eggs emerge to adults. Note that the fuzzy distribution model is based on qualitative arguments, however, the assumptions seem to be reasonable. Unfortunately, the settings of the required model parameters are not available from the literature. This would require case studies under different rainfall conditions as such from Paaijmans *et al. *[23]. In the second part of this study, parameter values of the fuzzy distribution model will be calibrated [14].

There are further possible future extensions of the LMM. For instance, a necessity for an improvement of the LMM concerns the dependence of immature development on water temperatures [126]. This would require the incorporation of meteorological variables such as potential evaporation, cloud cover, or sunshine duration [8] as well as information on their relation to water temperatures. The presented adult mosquito survival schemes are not fully satisfactory. Therefore, the incorporation of upcoming new information would be essential for future refinements of the LMM.

Ideally a more complete understanding of the complex ecology of malaria will require integration of research efforts across diverse areas [127]. The present formulation of the LMM actually misses various aspects of malaria. For example, the immune status of the human population as well as age-dependencies are neglected (cf. Figure 5). This would require the inclusion of the exposure history of individuals, which is not possible under the current mathematical formulation of the LMM. This issue of the LMM might be overcome by running the Garki model (see [5]) with *EIR *data from the LMM provided that the LMM_2010 _produces reasonable *EIR *values. The last fact will be shown by the second part of this study [14]. The hybrid LMM-Garki model was utilised by Ermert [13] and enables the simulation of realistic transmission rates between humans and mosquitoes (via the LMM_2010_) and provides a reasonable pattern of malaria exposure within the human population (via the Garki model).

## Conclusions

One of the most comprehensive studies to date in terms of gathering information from the malaria literature was undertaken for the development of a new version of an existing malaria model (Figures 4 and 5). An extensive literature survey with regard to entomological and parasitological malaria variables (see the seven Additional files) provided valuable information for a refined setting of most of the model parameters. This approach limits the degrees of freedom of the parameter space of the model simplifying the final calibration of undetermined parameters. In addition, the simulation of some key processes was changed in order to reflect a more physically correct relationship. For example, the oviposition as well as the survival of immature mosquitoes is now steered via a fuzzy distribution model. In the second part of this study [14], undetermined model parameters will be calibrated and the model simulations are validated by means of entomological and parasitological observations from West Africa.

## List of abbreviations

LMM: Liverpool Malaria Model; LMM_2004_:Liverpool Malaria Model version of 2004; LMM_2010_: Liverpool Malaria Model version of 2010; QT-NASBA: QuanTitative-Nucleic Acid Sequence-Based Amplification; RT-PCR: Reverse Transcriptase-Polymerase Chain Reaction. **List of symbols**: *EIR*: Entomological Inoculation Rate; *F*: fuzzy suitability; *groupC*: children between 2-10 years; *groupR*: rest of the population (excluding group C); *HBR*: Human Biting Rate; *n_m_*: maturation period of gametocytes; *n_mf_*: time needed for gametocytogenesis; *n_p_*: prepatent period; *PR*: Parasite Ratio; *R*_Σ10*d*_: 10-day accumulated rainfall; *sPR*: sexual Parasite Ratio; *T: *daily mean temperature.

## Competing interests

The authors declare that they have no competing interests.

## Authors' contributions

VE designed the study, undertook the literature review and proposed changes of the mathematical formulation of the LMM, as well as wrote the manuscript. AHF and APM contributed to the concept of the study. AHF supervised the PhD study of VE whose results formed the basis of the manuscript. APM was furthermore originally involved in the formulation of the LMM. AEJ contributed to the new design of LMM and provided the new model code. All authors read, suggested changes and approved the final manuscript.

## Supplementary Material

Additional file 1 **Number of produced eggs per *Anopheles *female**. Data with regard to the number of produced eggs per *Anopheles *female. Click here for file

Additional file 2 **Data in terms of the development of immature mosquitoes from vertical life tables**. Data in terms of the development immature mosquitoes taken from vertical life tables as derived under field conditions. Click here for file

Additional file 3 **Data regarding the development of immature mosquitoes from horizontal life tables**. Data regarding the development of immature mosquitoes taken from horizontal life tables as derived under controlled conditions. Click here for file

Additional file 4 **Mosquito survival probabilities**. Data with regard to the daily survival probability of adult mosquitoes (*p_d_*) as derived from entomological field studies. Click here for file

Additional file 5 **Mosquito-to-human transmission efficiencies**. Data regarding the mosquito-to-human transmission efficiency (*b*). Click here for file

Additional file 6 **Sexual parasite ratios**. Data with regard to the sexual parasite ratio (*sPR*), that is the percentage of humans with gametocytes in their blood as well as the ratio between sexual and asexual parasite ratio (*SAR*), which is the proportion of malaria parasite positive humans that are gametocytaemic. Click here for file

Additional file 7 **Human-to-mosquito transmission efficiencies**. Data with regard to the human-to-mosquito transmission efficiency (*c*), i.e. the proportion of mosquito bites on infectious humans which infect susceptible mosquitoes. Click here for file
