# Supplementary material for: Development of a new version of the Liverpool Malaria Model. I. Refining the parameter settings and mathematical formulation of basic processes based on a literature review
Source: Malar J. 2011 Feb 11;10:35. doi: 10.1186/1475-2875-10-35 (PMC3055220; doi:10.1186/1475-2875-10-35)
Supplement: Additional file 1 — Number of produced eggs per Anopheles female. Data with regard to the number of produced eggs per Anopheles female. [file 1475-2875-10-35-S1.PDF]

1 Number of produced eggs per Anopheles female

Data with regard to the number of produced eggs per *Anopheles* female.

Columns: country: country where the study was undertaken; place: location of the study site; long: longitude of the study site (-999.00: position is either unknown or was not seeked out); lat: latitude of the study site (-99.00: position is either unknown or was not seeked out); M1: month, when the study started; YYY1: year of the start of the study; M2: month, when the study ended; YYY2: year of the end of the study; $\#E_{p,ave}$: average number of produced eggs per female mosquito; $\#E_{p,min}$: minimum observed number of produced eggs; $\#E_{p,max}$: as $\#E_{p,min}$, but for the maximum; species: involved mosquito species; notes: notes; ref: reference. The '#' stands for 'number'. y denotes the number of produced eggs and x stands for the wing length (in mm). Minimum and maximum values refer to individual analysed mosquito females. The '-8' denotes data that were not available in the literature and that could not be checked due to limited access, respectively. Indices: [r]: the position of the study site was found in the reference.

country	place	long [°E]	lat [°N]	M1	YYY1	M2	YYY2	$\#E_{p,ave}$	$\#E_{p,min}$	$\#E_{p,max}$	species	notes	ref
Egypt	Faiyum	-999.00	-99.00	10	1983	-8	-8	-8.0	11.0	141.0	*An. sergentii*	laboratory	[1]
Egypt	Tersa	-999.00	-99.00	10	1990	11	1990	73.3	-8.0	-8.0	*An. pharoensis*	laboratory; # of eggs per female; constant temperatures ($25 \pm 2^{\circ}$C); $y = 73.3 \pm 16.9$	[2]
Egypt	Tersa	-999.00	-99.00	10	1990	11	1990	209.2	-8.0	-8.0	*An. pharoensis*	laboratory; # of eggs per female; cycling temperatures; $y = 209.2 \pm 36.8$	[2]
Egypt	Tersa	-999.00	-99.00	10	1990	11	1990	75.0	-8.0	-8.0	*An. multicolor*	laboratory; # of eggs per female; constant temperatures ($25 \pm 2^{\circ}$C); $y = 75.0 \pm 15.8$	[2]
Egypt	Tersa	-999.00	-99.00	10	1990	11	1990	164.8	-8.0	-8.0	*An. multicolor*	laboratory; # of eggs per female; cycling temperatures; $y = 164.8 \pm 61.5$	[2]
El Salvador	around Lake Apastepeque	-999.00	-99.00	06	1971	09	1972	120.0	-8.0	-8.0	*An. albimanus*	-	[3]
Gambia, The	Kaba Kamma	-999.00	-99.00	07	1993	08	1993	-8	20.0	180.0	*An. gambiae* s.s.	laboratory; # of laid and retained eggs; indoor resting females; $y = 46.67x - 56.7$, x range: 2.5-3.3 mm	[4]
Gambia, The	Kaba Kamma	-999.00	-99.00	07	1993	08	1993	-8	5.0	160.0	*An. arabiensis*	laboratory; # of laid and retained eggs; indoor resting females; $y = 66.72x - 125.68$; x range:. 2.7-3.3 mm	[4]
Tanzania	Kilimanjaro region	-999.00	-99.00	-8	-8	-8	-8	12.6	-8.0	-8.0	*An. gambiae* s.s.	laboratory; # of mature oocytes; small females; blood meal size: 1.0 μl	[5]
Tanzania	Kilimanjaro region	-999.00	-99.00	-8	-8	-8	-8	108.6	-8.0	-8.0	*An. gambiae* s.s.	laboratory; # of mature oocytes; large females; blood meal: rat once	[5]
Tanzania	Michenga	36.63	8.17[r]	-8	1991	-8	1991	150.0	66.0	290.0	*An. gambiae* s.l.	laboratory; # of laid and retained eggs; indoor resting females; $y = 133.93x - 187.00$ (note: the value of the y-intercept disagrees with the inserted line in Fig. 3 in the reference); x range: 2.5-3.3 mm	[6]
Tanzania	Michenga	36.63	8.17[r]	-8	1991	-8	1991	111.0	48.0	178.0	*An. gambiae* s.l.	laboratory; # of laid and retained eggs; newly emerged females; $y = 89.13x - 152.96$	[6]

References

1. Beier MS, Beier JC, Merdan AA, Sawaf BME, Kadder MA: **Laboratory rearing techniques and adult life table parameters for *Anopheles sergentii* from Egypt.** *J Am Mosq Control Asso* 1987, **3**:266–270.
2. Kenawy MA: **Development and survival of *Anopheles pharoensis* and *An. multicolor* from Faiyum, Egypt.** *J Am Mosq Control Assoc* 1991, **7**:551–555.
3. Weidhaas DE, Breeland SG, Lofgren CS, Dame DA, Kaiser R: **Release of chemosterilized males for the control of *Anopheles Albimanus* in El Salvador. IV. Dynamics of the test population.** *Am J Trop Med Hyg* 1974, **23**:298–308.
4. Hogg JC, Thompson MC, Hurd H: **Comparative fecundity and associated factors for two sibling species of the *Anopheles gambiae* complex occuring sympatrically in The Gambia.** *Med Vet Entomol* 1996, **10**:385–391.
5. Takken W, Klowden MJ, Chambers GM: **Effect of body size on host seeking and blood meal utilization in *Anopheles gambiae sensu stricto* (Diptera: Culicidae): the disadvantage of being small.** *J Med Entomol* 1998, **35**:639–645.
6. Lyimo EO, Takken W: **Effects of adult body size on fecundity and the pre-gravid rate of *Anopheles gambiae* females in Tanzania.** *Med Vet Entomol* 1993, **7**:328–332.
