# Supplementary material for: Development of a new version of the Liverpool Malaria Model. I. Refining the parameter settings and mathematical formulation of basic processes based on a literature review
Source: Malar J. 2011 Feb 11;10:35. doi: 10.1186/1475-2875-10-35 (PMC3055220; doi:10.1186/1475-2875-10-35)
Supplement: Additional file 2 — Data in terms of the development of immature mosquitoes from vertical life tables. Data in terms of the development immature mosquitoes taken from vertical life tables as derived under field conditions. [file 1475-2875-10-35-S2.PDF]

2 Data in terms of the development of immature mosquitoes from vertical life tables

Data in terms of the development of immature mosquitoes taken from vertical life tables as derived under field conditions.

Columns: country: country where the study was undertaken; place: location of the study site; long: longitude of the study site (-999.00: position is either unknown or was not seeked out); lat: latitude of the study site (-99.00: position is either unknown or was not seeked out); M1: month, when the study started; YYY1: year of the start of the study; M2: month, when the study ended; YYY2: year of the end of the study; $p_{MMA,ave}$: averaged survival probability of immature mosquitoes from egg to adult emergence, that is the proportion of eggs reaching MMA; $p_{MMA,min}$: as $p_{MMA,ave}$, but for the minimum; $p_{MMA,max}$: as $p_{MMA,ave}$, but for the maximum; MMA: mosquito mature age, that is the duration between egg laying and adult emergence; $\eta_{d,ave}$: average daily survival probability of immature mosquitoes; $\eta_{d,ave}$: as $\eta_{d,min}$, but for the minimum; $\eta_{d,max}$; as $\eta_{d,ave}$, but for the maximum; species: involved mosquito species; notes: notes; ref: reference. The '-8' and '-9' denote data that were not available in the literature and that could not be checked due to limited access, respectively. Indices: ^{r}: the position of the study site was found in the reference.

country	place	long [°E]	lat [°N]	M1	YYY1	M2	YYY2	$p_{MMA,ave}$ [%]	$p_{MMA,min}$ [%]	$p_{MMA,max}$ [%]	MMA [days]	$\eta_{d,ave}$ [%]	$\eta_{d,min}$ [%]	$\eta_{d,max}$ [%]	species	notes	ref
El Salvador	Lake Apastepeque	-999.00	-99.00	06	1971	09	1972	4.8	2.0	15.0	-8.0	-8.0	-8.0	-8.0	*An. albimanus*	-	[1]
Kenya	Chiga	-999.00	-99.00	-8	-8	-8	-8	-8.0	3.8	4.8	12.5	-8.0	77.0	78.4	*An. gambiae*	marsh and pool collections	[2]
Kenya	Ahero, Rabour, Nduru	34.75	-0.1^r	07	1974	07	1974	-8.0	6.6	7.4	11.8	-8.0	79.4	80.2	*An. gambiae*	rice field, pond, and pool collections; $z = 1000$	[3]
Kenya	Ahero	34.75	-0.1^r	07	1974	08	1974	16.5	-8.0	-8.0	11.8	85.8	-8.0	-8.0	*An. gambiae*	sprayed rice field collections; $z = 1000$	[3]
Kenya	Chiga, Rabour, Kanyamedha, Warthorego	-999.00	-99.00	11	1971	12	1971	-8.0	2.9	3.4	11.8	-8.0	74.0	75.0	*An. gambiae*	pond collections; $z = 1150$	[4]
Kenya	Chiga	-999.00	-99.00	11	1971	12	1971	0.0	-8.0	-8.0	11.8	-8.0	-8.0	-8.0	*An. gambiae*	ditch collections; $z = 1150$	[4]
Kenya	Mwea	-999.00	-99.00	08	2005	04	2006	1.7	0.1	3.4	11.9	69.4	52.7	75.2	*An. arabiensis*	rice field collections; different rice stages (land preparation, transplanting, and tillering)	[5]
Mali	Banambani	-8.05	12.80^r	07	2000	08	2000	17.6	8.0	28.5	11.8	86.3	80.7	89.9	*An. gambiae* s.l.	rock pool, swamp, and puddle collections	[6]
Kenya	Baringo	-999.00	-99.00	-9	-9	-9	-9	-9.0	-9.0	-9.0	-9.0	91.9	-9.0	-9.0	*An. gambiae*	-	[7]

References

1. Weidhaas DE, Breeland SG, Lofgren CS, Dame DA, Kaiser R: **Release of chemosterilized males for the control of *Anopheles Albimanus* in El Salvador. IV. Dynamics of the test population.** *Am J Trop Med Hyg* 1974, **23**:298–308.
2. Service MW: **Studies on sampling larval populations of the *Anopheles gambiae* complex.** *Bull World Health Org* 1971, **45**:169–180.
3. Service MW: **Mortalities of the immature stages of species B of the *Anopheles gambiae* complex in Kenya: comparison between rice fields and temporary pools, identification of predators, and effects of insecticidal spraying.** *J Med Entomol* 1977, **13**:535–545.
4. Service MW: **Mortalities of the larvae of the *Anopheles gambiae* Giles complex and detection of predators by the precipitin test.** *Bull Entomol Res* 1973, **62**:359–369.
5. Mwangangi JM, Muturi EJ, Shililu J, Muriu SM, Jacob B, Kabiru EW, Mbogo CM, Githure J, Novak R: **Survival of immature *Anopheles arabiensis* (Diptera: Culicidae) in aquatic habitats in Mwea rice irrigation scheme, Central Kenya.** *Malar J* 2006, **5**:114.
6. Edillo FE, Touré YT, Lanzaro GC, Dolo G, Taylor CE: **Survivorship and distribution of immature *Anopheles gambiae s.l.* (Diptera: Culicidae) in Banambani village, Mali.** *J Med Entomol* 2004, **41**:333–339.
7. Aniedu I, Mutinga MJ, Mutero CM: **Vertical estimates of survivorship of larvae and pupae of *Anopheles gambiae* Giles complex in Baringo district, Kenya.** *Insect Science and its Application* 1993, **14**:39–48.
