# Supplementary material for: Development of a new version of the Liverpool Malaria Model. I. Refining the parameter settings and mathematical formulation of basic processes based on a literature review
Source: Malar J. 2011 Feb 11;10:35. doi: 10.1186/1475-2875-10-35 (PMC3055220; doi:10.1186/1475-2875-10-35)
Supplement: Additional file 3 — Data regarding the development of immature mosquitoes from horizontal life tables. Data regarding the development of immature mosquitoes taken from horizontal life tables as derived under controlled conditions. [file 1475-2875-10-35-S3.PDF]

3 Data regarding the development of immature mosquitoes from horizontal life tables

Data regarding the development of immature mosquitoes taken from horizontal life tables as derived under controlled conditions.

Columns: country: country where the study was undertaken; place: location of the study site; long: longitude of the study site (-999.00: position is either unknown or was not seeked out); lat: latitude of the study site (-99.00: position is either unknown or was not seeked out); M1: month, when the study started; YYY1: year of the start of the study; M2: month, when the study ended; YYY2: year of the end of the study; $p_{MMA,ave}$: averaged immature survival probability from egg to adult emergence, that is the proportion of immature mosquitoes reaching the mosquito mature age; $p_{MMA,min}$: as $p_{MMA,ave}$, but for the minimum; $p_{MMA,max}$: as $p_{MMA,ave}$, but for the maximum; MMA_{ave}: average mosquito mature age; MMA_{min}: minimum mosquito mature age; MMA_{max}: maximum mosquito mature age; $\eta_{dc,ave}$: average daily immature mosquito survival probability in protected habitats; $\eta_{dc,min}$: as $\eta_{dc,ave}$, but for the minimum; $\eta_{dc,max}$: as $\eta_{dc,ave}$, but for the maximum; species: involved mosquito species; notes: notes; ref: reference. The '-8' denotes data that were not available in the literature and that could not be checked due to limited access, respectively. Note that miminum and maximum values refer to average values obtained by different experimental settings (T: different temperatures; ρ: different larval densities; Dl: different day lengths; Ñ: with additional nutrients; ¬Ñ: without added nutrients). Particular applied experimental settings are specified under the 'note' column. Indices: ^{r}: the position of the study site was found in the reference; ^{p}: time to pupation; ^{e}: time to adult emergence.

country	place	long [°E]	lat [°N]	M1	YYY1	M2	YYY2	$p_{MMA,ave}$	$p_{MMA,min}$	$p_{MMA,max}$	MMA_{ave}	MMA_{min}	MMA_{max}	$\eta_{dc,ave}$	$\eta_{dc,min}$	$\eta_{dc,max}$	species	notes	ref
Egypt	Faiyum	-999.00	-99.00	10	1983	-8	-8	-8.0	55.8e	72.3e	-8.0	14.2	33.2	-8.0	97.7	98.2	*An. sergentii*	laboratory; T: 17/(22)/27°C; mud slurry, lower survival in tab/distilled water	[1]
Egypt	Tersa	-999.00	-99.00	10	1990	11	1990	13.0	-8.0	-8.0	$16.5^{p}/18.5^{e}$	-8.0	-8.0	89.6	-8.0	-8.0	*An. pharoensis*	laboratory	[2]
Egypt	Tersa	-999.00	-99.00	10	1990	11	1990	22.0	-8.0	-8.0	$20.0^{p}/21.0^{e}$	-8.0	-8.0	93.0	-8.0	-8.0	*An. multicolor*	laboratory	[2]
Kenya	western Kenyan highland area	-999.00	-99.00	09	2003	09	2003	-8.0	24.0	48.0	-8.0	11.2^{e}	16.5^{e}	-8.0	91.7	94.9	*An. gambiae s.l.*	farmland; ρ ¬Ñ; $z = 1420$-1580	[3]
Kenya	western Kenyan highland area	-999.00	-99.00	09	2003	09	2003	0.0	0.0	0.0	-8.0	-8.0	-8.0	-8.0	-8.0	-8.0	*An. gambiae s.l.*	forest; ρ ¬Ñ; $z = 1420$-1580	[3]
Kenya	western Kenyan highland area	-999.00	-99.00	09	2003	09	2003	0.0	0.0	0.0	-8.0	-8.0	-8.0	-8.0	-8.0	-8.0	*An. gambiae s.l.*	swamp; ρ ¬Ñ; $z = 1420$-1580	[3]
Kenya	western Kenyan highland area	-999.00	-99.00	06	2004	06	2004	-8.0	49.0	65.0	-8.0	12.0^{e}	16.7^{e}	-8.0	95.3	96.5	*An. gambiae s.l.*	farmland; ρ ¬Ñ; $z = 1420$-1580	[3]
Kenya	western Kenyan highland area	-999.00	-99.00	06	2004	06	2004	-8.0	0.0	2.0	-8.0	20.1^{e}	27.6^{e}	-8.0	82.3	86.8	*An. gambiae s.l.*	forest; ρ ¬Ñ; $z = 1420$-1580	[3]
Kenya	western Kenyan highland area	-999.00	-99.00	06	2004	06	2004	-8.0	6.0	33.0	-8.0	20.1^{e}	27.6^{e}	-8.0	88.7	94.5	*An. gambiae s.l.*	swamp; ρ ¬Ñ; $z = 1420$-1580	[3]
Kenya	western Kenyan highland area	-999.00	-99.00	06	2004	06	2004	-8.0	61.0	70.0	-8.0	9.0^{e}	12.1^{e}	-8.0	95.8	96.1	*An. gambiae s.l.*	farmland; ρ Ñ; $z = 1420$-1580	[3]
Kenya	western Kenyan highland area	-999.00	-99.00	06	2004	06	2004	-8.0	10.0	23.0	-8.0	18.4^{e}	24.2^{e}	-8.0	90.8	93.0	*An. gambiae s.l.*	forest; ρ Ñ; $z = 1420$-1580	[3]
Kenya	western Kenyan highland area	-999.00	-99.00	06	2004	06	2004	-8.0	24.0	43.0	-8.0	19.3^{e}	23.7^{e}	-8.0	94.2	96.2	*An. gambiae s.l.*	swamp; ρ Ñ; $z = 1420$-1580	[3]
Kenya	near Kisumu	-999.00	-99.00	-8	-8	-8	-8	-8.0	53.5	60.6	-8.0	8.5^{p}	9.9^{p}	-8.0	93.9	94.3	*An. gambiae*	ρ ¬Ñ;	[4]
Kenya	near Kisumu	-999.00	-99.00	-8	-8	-8	-8	-8.0	49.4	60.0	-8.0	8.1^{p}	10.3^{p}	-8.0	91.6	95.2	*An. gambiae*	ρ Ñ;	[4]
Kenya	Fort Ternan	35.35	-0.20^{r}	06	2001	08	2001	0.4	-8.0	-8.0	16.5^{p}	-8.0	-8.0	71.6	-8.0	-8.0	*An. gambiae s.s.*, *An. arabiensis*	$z = 1550$-1650	[5]

to be continued

Table 1 – continued

country	place	long [°E]	lat [°N]	M1	YYY1	M2	YYY2	$p_{MMA,ave}$	$p_{MMA,min}$	$p_{MMA,max}$	MMA_{ave}	MMA_{min}	MMA_{max}	$\eta_{dc,ave}$	$\eta_{dc,min}$	$\eta_{dc,max}$	species	notes	ref
Liberia	Sua	-999.00	-99.00	-8	-8	-8	-8	-8.0	55.0	80.0	-8.0	10.6[p]	11.6[p]	-8.0	94.5	98.1	*An. gambiae* s.s.	laboratory; ρ	[6]
Liberia	-	-999.00	-99.00	-8	-8	-8	-8	83.4	72.0	99.0	9.8[p]	8.0[p]	13.0	98.2	95.8	99.9	*An. gambiae* s.s.	laboratory; T ρ	[7]
Nigeria	Lagos	-999.00	-99.00	-8	-8	-8	-8	-8.0	-8.0	-8.0	-8.0	-8.0	-8.0	-8.0	88.9	98.9	*An. gambiae* s.s.	laboratory; T; no maturation at $T < 18°$C and $T > 32°$C	[8]
Mali	Banambani	-8.05	12.80[r]	07	2000	08	2000	68.5	-8.0	-8.0	11.8[e]	-8.0	-8.0	96.8	-8.0	-8.0	*An. gambiae* s.l.	laboratory	[9]
Zimbabwe	Zambezi valley	-999.00	-99.00	-8	-8	-8	-8	-8.0	37.0	65.0	-8.0	9.9[p]	10.4[p]	-8.0	90.4	96.0	*An. arabiensis*	laboratory; ρ	[6]
-	-	-999.00	-99.00	-8	-8	-8	-8	-8.0	35.0	85.0	-8.0	5.4[e]	30.0[e]	-8.0	94.8	98.9	*Culex pipiens* complex	laboratory; T Dl	[10]

References

1. Beier MS, Beier JC, Merdan AA, Sawaf BME, Kadder MA: **Laboratory rearing techniques and adult life table parameters for *Anopheles sergentii* from Egypt.** *J Am Mosq Control Asso* 1987, **3**:266–270.
2. Kenawy MA: **Development and survival of *Anopheles pharoensis* and *An. multicolor* from Faiyum, Egypt.** *J Am Mosq Control Assoc* 1991, **7**:551–555.
3. Munga S, Minakawa N, Zhou G, Mushinzimana E, Barrack OOJ, Githeko AK, Yan G: **Association between land cover and habitat productivity of malaria vectors in western Kenyan highlands.** *Am J Trop Med Hyg* 2006, **74**:69–75.
4. Gimnig JE, Ombok M, Otieno S, Kaufman MG, Vulule JM, Walker ED: **Density-dependent development of *Anopheles gambiae* (Diptera: Culicidae) larvae in artificial habitats.** *J Med Entomol* 2002, **39**:162–172.
5. Koenraadt CJM, Paaijmans KP, Schneider P, Githeko AK, Takken W: **Low larval vector survival explains unstable malaria in the western Kenya highlands.** *Trop Med Int Health* 2006, **11**:1195–1205.
6. Schneider P, Takken W, McCall PJ: **Interspecific competition between sibling species larvae of *Anopheles arabiensis* and *An. gambiae*.** *Med Vet Entomol* 2000, **14**:165–170.
7. Lyimo EO, Takken W, Koella J: **Effect of rearing temperature and larval density on larval survival, age at pupation and adult size of *Anopheles gambiae*.** *Entomol Exp Appl* 1992, **63**:265–271.
8. Bayoh MN, Lindsay SW: **Temperature-related duration of aquatic stages of the Afrotropical malaria vector mosquito *Anopheles gambiae* in the laboratory.** *Med Vet Entomol* 2004, **18**:174–179.
9. Edillo FE, Touré YT, Lanzaro GC, Dolo G, Taylor CE: **Survivorship and distribution of immature *Anopheles gambiae s.l.* (Diptera: Culicidae) in Banambani village, Mali.** *J Med Entomol* 2004, **41**:333–339.
10. Mogi M: **Temperature and photoperiod effects on larval ovarian development of New Zealand strains of *Culex quinquefasciatus* (Diptera: Culicidae).** *Ann Entomol Soc Am* 1992, **85**:58–66.
