# Supplementary material for: Development of a new version of the Liverpool Malaria Model. I. Refining the parameter settings and mathematical formulation of basic processes based on a literature review
Source: Malar J. 2011 Feb 11;10:35. doi: 10.1186/1475-2875-10-35 (PMC3055220; doi:10.1186/1475-2875-10-35)
Supplement: Additional file 4 — Mosquito survival probabilities. Data with regard to the daily survival probability of adult mosquitoes (pd) as derived from entomological field studies. [file 1475-2875-10-35-S4.PDF]

# 4 Mosquito survival probabilities

Data with regard to the daily survival probability of adult mosquitoes ($p_d$) as derived from entomological field studies.

Columns: country: country where the study was undertaken; place: location of the study site; long: longitude of the study site (-999.00: position is either unknown or was not seeked out); lat: latitude of the study site (-99.00: position is either unknown or was not seeked out); M1: month, when the study started; YYY1: year of the start of the study; M2: month, when the study ended; YYY2: year of the end of the study; $p_{d,ave}$: average value of $p_d$; $p_{d,min}$: minimum of $p_d$; $p_{d,max}$: maximum of $p_d$; way: determination way of $p_d$ (C: calculated/estimated, see corresponding paper; K: keeping in cages/laboratory; A: age-grading [1]; P: parous rate; S: immediate and delayed sporozoite rates [2]; L: ampulla measurements; M: mark-release-recapture method; I: population decline during the dry period [3]); species: involved mosquito species; wea: weather conditions during the experiment (R: rainy season; D: dry season; T: transition season between either dry and rainy or rainy and dry season; E: dry and rainy season); notes: notes; ref: reference. The '-8' and '-9' denote data that were not available in the literature and that could not be checked due to limited access, respectively. Minimum and maximum values refer to annual variations. Indices: [a]: the position of the study site was taken from Hay *et al.* [4]; [b]: the position of the study site was extracted from Hay *et al.* [5]; [r]: the position of the study site was found in the reference.

| country | place | long [°E] | lat [°N] | M1 | YYY1 | M2 | YYY2 | $p_{d,ave}$ | $p_{d,min}$ | $p_{d,max}$ | way | species | wea | notes | ref |
|---|---|---|---|---|---|---|---|---|---|---|---|---|---|---|---|
| Brasil | Pariquera-Açu county | -47.83 | -24.50 | 01 | 2000 | 02 | 2000 | 61.0 | -8.0 | -8.0 | M | *An. albitarsis* | R | - | [6] |
| Burkina Faso | Bobo-Dioulasso | -999.00 | -99.00 | 06 | 1959 | 12 | 1960 | 88.3 | -8.0 | -8.0 | P | *An. gambiae* | - | - | [7] |
| Burkina Faso | Bobo-Dioulasso | -999.00 | -99.00 | 06 | 1959 | 12 | 1960 | 90.7 | -8.0 | -8.0 | P | *An. funestus* | - | - | [7] |
| Burkina Faso | Bobo-Dioulasso | -999.00 | -99.00 | 06 | 1959 | 12 | 1960 | 84.2 | -8.0 | -8.0 | P | *An. nili* | - | - | [7] |
| Burkina Faso | Bobo-Dioulasso | -999.00 | -99.00 | 06 | 1959 | 12 | 1960 | 88.9 | -8.0 | -8.0 | P | *An. coustani* | - | - | [7] |
| Burkina Faso | Bobo-Dioulasso, urban | -999.00 | -99.00 | 01 | 1985 | 12 | 1985 | 71.0 | -8.0 | -8.0 | P | *An. gambiae* | R | - | [8] |
| Burkina Faso | Bobo-Dioulasso, rural | -999.00 | -99.00 | -8 | -8 | -8 | -8 | 91.0 | -8.0 | -8.0 | P | *An. gambiae* | R | - | [8] |
| Burkina Faso | Goundri | -1.33 | 12.50[r] | 09 | 1991 | 09 | 1991 | 73.6 | -8.0 | -8.0 | M | mixture of *Anopheles* | R | other estimates provide higher values | [9] |
| Burkina Faso | Goundri | -1.33 | 12.50[r] | 09 | 1992 | 09 | 1992 | 74.3 | -8.0 | -8.0 | M | *An. gambiae s.l.* | R | other estimates provide higher values | [9] |
| Cameroon | Gounougou | -999.00 | -99.00 | 07 | 1990 | 09 | 1990 | 68.0 | -8.0 | -8.0 | P | *An. gambiae s.l.* | R | - | [10] |
| Cameroon | Gounougou | -999.00 | -99.00 | 07 | 1990 | 09 | 1990 | 79.0 | -8.0 | -8.0 | P | *An. funestus* | R | - | [10] |
| Cameroon | Gounougou | -999.00 | -99.00 | 07 | 1990 | 09 | 1990 | 62.0 | -8.0 | -8.0 | P | *An. pharoensis* | R | - | [10] |
| Côte d'Ivoire | Alloukoukro | -5.08 | 7.80[b] | 01 | 1991 | 12 | 1991 | 87.0 | 80.0 | 97.0 | P | *An. gambiae s.l.* | E | - | [11] |
| Côte d'Ivoire | Alloukoukro | -5.08 | 7.80[b] | 01 | 1992 | 12 | 1992 | 89.0 | 82.0 | 95.0 | P | *An. gambiae s.l.* | E | - | [11] |
| Côte d'Ivoire | Alloukoukro | -5.08 | 7.80[b] | 01 | 1992 | 12 | 1992 | 90.0 | 84.0 | 100.0 | P | *An. funestus* | E | - | [11] |
| Côte d'Ivoire | Alloukoukro | -5.08 | 7.80[b] | 01 | 1992 | 12 | 1992 | 91.0 | 87.0 | 100.0 | P | *An. funestus* | E | - | [11] |
| Dominican Republic | Calle Duarte, Colonia Japonesa, La Bomba | -999.00 | -99.00 | 07 | 1987 | 10 | 1988 | 68.4 | -8.0 | -8.0 | P | *An. albimanus* | - | - | [12] |
| Dominican Republic | Calle Duarte, Colonia Japonesa, La Bomba | -999.00 | -99.00 | 07 | 1987 | 10 | 1988 | 61.1 | -8.0 | -8.0 | P | *An. vestitipennis* | - | - | [12] |
| Egypt | Faiyum | -999.00 | -99.00 | -9 | -9 | -9 | -9 | 95.0 | -8.0 | -8.0 | K | *An. pharoensis* | - | - | [13] |
| Egypt | Faiyum | -999.00 | -99.00 | -9 | -9 | -9 | -9 | 93.0 | -8.0 | -8.0 | K | *An. multicolor* | - | - | [13] |
| Egypt | Faiyum | -999.00 | -99.00 | -9 | -9 | -9 | -9 | 89.0 | -8.0 | -8.0 | P | *An. pharoensis* | - | - | [13] |
| Egypt | Faiyum | -999.00 | -99.00 | -9 | -9 | -9 | -9 | 80.0 | -8.0 | -8.0 | P | *An. multicolor* | - | - | [13] |
| Egypt | Faiyum | -999.00 | -99.00 | 10 | 1983 | -8 | -8 | 95.0 | -8.0 | -8.0 | K | *An. sergentii* | - | laboratory; $T$: $27 \pm 2°C$ | [14] |
| El Salvador | around Lake Apastepeque | -999.00 | -99.00 | 01 | 1972 | 04 | 1972 | 67.5 | 65.0 | 70.0 | M | *An. albimanus* | D | MRR/release of sterile females | [15] |
| El Salvador | around Lake Apastepeque | -999.00 | -99.00 | 06 | 1971 | 08 | 1972 | 82.4 | 73.0 | 91.0 | M | *An. albimanus* | R | MRR/release of sterile females; data for May-Sep. | [15] |
| El Salvador | around Lake Apastepeque | -999.00 | -99.00 | 06 | 1971 | 08 | 1972 | 77.7 | 65.0 | 91.0 | M | *An. albimanus* | R/D | MRR/release of sterile females | [15] |
| Iran | Arso | -999.00 | -99.00 | -8 | -8 | -8 | -8 | 85.5 | -8.0 | -8.0 | P | *An. koliensis* | - | reduced under DDT | [7] |
| Iran | Arso | -999.00 | -99.00 | -8 | -8 | -8 | -8 | 89.5 | -8.0 | -8.0 | P | *An. farauti* | - | reduced under DDT | [7] |
| Iran | Arso | -999.00 | -99.00 | -8 | -8 | -8 | -8 | 84.5 | -8.0 | -8.0 | P | *An. punctulatus* | - | - | [7] |

to be continued

**Table 1 – continued**

| country | place | long [°E] | lat [°N] | M1 | YYY1 | M2 | YYY2 | $p_{d,ave}$ | $p_{d,min}$ | $p_{d,max}$ | way | species | wea | notes | ref |
|---|---|---|---|---|---|---|---|---|---|---|---|---|---|---|---|
| Iran | Baluchistan | -999.00 | -99.00 | 05 | 1991 | 10 | 1991 | -8.0 | 84.0 | 89.0 | -9 | *An. culicifacies* s.l. | - | $p_d$ is lower in sprayed villages | [16] |
| Iran | Baluchistan | -999.00 | -99.00 | 05 | 1991 | 10 | 1991 | -8.0 | 80.0 | 83.0 | -9 | *An. pulcherrimus* | - | $p_d$ is lower in sprayed villages | [16] |
| Kenya | Mgandini | -999.00 | -99.00 | 10 | 1972 | 11 | 1972 | 89.0 | -8.0 | -8.0 | M | *Aedes aegypti* | R | - | [17] |
| Kenya | Msihu | 39.28 | -4.53[r] | 03 | 1984 | 04 | 1984 | 80.9 | -8.0 | -8.0 | C | *An. gambiae* s.l. | D | - | [18] |
| Kenya | Msihu | 39.28 | -4.53[r] | 05 | 1982 | 06 | 1982 | 71.4 | -8.0 | -8.0 | C | *An. gambiae* s.l. | R | - | [18] |
| Kenya | Jimbo | 39.23 | -4.67[r] | 11 | 1982 | 12 | 1982 | 74.1 | -8.0 | -8.0 | C | *An. merus* | R | - | [18] |
| Kenya | Mwea | -999.00 | -99.00 | 08 | 1983 | 09 | 1983 | 81.0 | -8.0 | -8.0 | C | *An. arabiensis* | R | - | [18] |
| Korea | Paju | -999.00 | -99.00 | 06 | 2000 | 08 | 2000 | 78.7 | 70.6 | 87.2 | P | *An. sinensis* | - | - | [19] |
| Korea | Kyonggi-do, malarious area | -999.00 | -99.00 | 06 | 2000 | 08 | 2000 | 86.4 | 80.4 | 90.0 | P | *An. sinensis* | - | - | [20] |
| Korea | Kyonggi-do, non-malarious area | -999.00 | -99.00 | 06 | 2000 | 08 | 2000 | 83.3 | 80.7 | 86.6 | P | *An. sinensis* | - | - | [20] |
| Korea | Gyonggi-do | -999.00 | -99.00 | 06 | 1999 | 10 | 1999 | 89.0 | 84.3 | 92.2 | P | *An. sinensis* | - | large variation | [21] |
| Korea | Gyeonggi Province | 126.83 | 37.93[r] | 04 | 1999 | 10 | 1999 | 85.9 | 80.4 | 89.5 | P | *An. sinensis* | - | - | [22] |
| Mexico | Chiapas, coastal plain | -999.00 | -99.00 | -9 | -9 | -9 | -9 | -9.0 | 45.0 | 58.0 | -9 | *An. vestitipennis* | - | - | [23] |
| Mexico | Chiapas, Lacandon Forest | -999.00 | -99.00 | -9 | -9 | -9 | -9 | 68.0 | -9.0 | -9.0 | -9 | - | - | *An. vestitipennis* | [23] |
| Mexico | Tapachula foothills | -999.00 | -99.00 | -9 | -9 | -9 | -9 | -9.0 | 87.5 | 88.4 | P | *An. pseudopunctipennis* | D | - | [24] |
| Nigeria | Kankiya | 7.83 | 12.55 | 06 | 1967 | 10 | 1967 | 93.8 | -8.0 | -8.0 | P | *An. gambiae* species B | R | reduced under DDT | [25] |
| Nigeria | Kaduna area | -999.00 | -99.00 | 05 | 1963 | 08 | 1963 | 90.0 | -8.0 | -8.0 | S | *An. gambiae* | R | - | [26] |
| Nigeria | Kaduna area | -999.00 | -99.00 | 05 | 1963 | 08 | 1963 | 89.0 | -8.0 | -8.0 | S | *An. funestus* | R | - | [26] |
| Pakistan | 12 villages around Khagrachberi | -999.00 | -99.00 | 07 | 1966 | 06 | 1967 | 90.0 | -8.0 | -8.0 | P | *An. minimus* | - | - | [27] |
| Pakistan | 12 villages around Khagrachberi | -999.00 | -99.00 | 07 | 1966 | 06 | 1967 | 86.0 | -8.0 | -8.0 | P | *An. vagus* | - | - | [27] |
| Pakistan | 12 villages around Khagrachberi | -999.00 | -99.00 | 07 | 1966 | 06 | 1967 | 81.0 | -8.0 | -8.0 | P | *An. jeyporiensis* | - | - | [27] |
| Pakistan | 12 villages around Khagrachberi | -999.00 | -99.00 | 07 | 1966 | 06 | 1967 | 72.0 | -8.0 | -8.0 | P | *An. philippinensis* | - | - | [27] |
| Pakistan | Sattoki | -999.00 | -99.00 | 05 | 1977 | 05 | 1977 | 80.8 | -8.0 | -8.0 | M | *An. stephensi* | R | - | [28] |
| Papua New Guinea | Butelgut | -999.00 | -99.00 | -8 | -8 | -8 | -8 | 86.0 | -8.0 | -8.0 | -9 | *An. punctulatus* | - | calculated via a method from [29] | [30] |
| Senegal | Aéré Lao | -14.30 | 16.40[b] | 09 | 1982 | 12 | 1982 | -8.0 | 93.0 | 97.0 | P | *An. gambiae* s.l. | - | - | [31] |
| Senegal | Barkedji | -14.87 | 15.28[b] | -8 | 1993 | -8 | 1993 | 86.8 | -8.0 | -8.0 | C | *Aedes vexans arabiensis* | R | - | [32] |
| Senegal | Boké Diallobé | -14.00 | 16.10[b] | 09 | 1982 | 11 | 1983 | -8.0 | 90.0 | 94.0 | P | *An. gambiae* s.l. | - | - | [31] |
| Senegal | Pikine | -17.40 | 14.75[b] | 12 | 1979 | 12 | 1980 | 82.2 | 73.8 | 90.5 | P | *An. arabiensis* | - | $p_d$ (rainy season) > $p_d$ (dry season) | [33] |
| Senegal | Pikine | -17.40 | 14.75[b] | 10 | 1979 | 12 | 1980 | 85.8 | -8.0 | -8.0 | C | - | - | - | [34] |
| Senegal | Pikine | -17.40 | 14.75[b] | 12 | 1981 | 12 | 1982 | 82.0 | 77.0 | 84.0 | C | *An. arabiensis* | - | $p_d$ (rainy season) > $p_d$ (dry season) | [34] |
| Sierra Leone | Bayama | -11.77 | 8.00[a] | -9 | -9 | -9 | -9 | 85.0 | -9.0 | -9.0 | -9 | *An. gambiae* | - | - | [35] |
| Sri Lanka | - | -999.00 | -99.00 | 06 | 1983 | 05 | 1984 | 93.8 | -8.0 | -8.0 | K | *An. culicifacies* | - | - | [36] |
| Sudan | Ed dekeinat | -999.00 | -99.00 | 10 | 1995 | 12 | 1996 | -8.0 | 70.7 | 84.9 | P | *An. arabiensis* | R/T | - | [37] |
| Sudan | El manshial | -999.00 | -99.00 | 10 | 1995 | 12 | 1996 | -8.0 | 69.3 | 80.6 | P | *An. arabiensis* | R/T | - | [37] |
| Sudan | Ed dekeinat | -999.00 | -99.00 | 10 | 1995 | 12 | 1996 | 57.4 | -8.0 | -8.0 | P | *An. arabiensis* | D | - | [37] |
| Sudan | El manshial | -999.00 | -99.00 | 10 | 1995 | 12 | 1996 | 61.6 | -8.0 | -8.0 | P | *An. arabiensis* | D | - | [37] |
| Tanzania | Muheza | -999.00 | -99.00 | 02 | 1963 | 12 | 1963 | 87.6 | 85.8 | 90.6 | P | *An. gambiae* species A | - | - | [25, 38] |
| Tanzania | Muheza | -999.00 | -99.00 | 05 | 1963 | 08 | 1963 | 85.1 | 82.5 | 89.4 | P | *An. gambiae* species A | - | - | [25, 38] |
| Tanzania | Muheza | -999.00 | -99.00 | 01 | 1963 | 12 | 1963 | 86.1 | 83.9 | 88.2 | P | *An. gambiae* species A | - | - | [25, 38] |
| Tanzania | Muheza | -999.00 | -99.00 | 11 | 1962 | 12 | 1963 | 89.4 | 85.0 | 92.0 | P | *An. funestus* | - | - | [25, 38] |
| Tanzania | Gonja | -999.00 | -99.00 | 12 | 1962 | 01 | 1964 | 82.6 | 77.9 | 87.3 | P | *An. gambiae* s.l. | - | - | [25, 38] |
| Tanzania | - | -999.00 | -99.00 | -8 | 1952 | -8 | 1952 | 91.0 | -8.0 | -8.0 | P | *An. gambiae* | - | - | [39] |
| Tanzania | - | -999.00 | -99.00 | -8 | 1952 | -8 | 1952 | 93.0 | -8.0 | -8.0 | S | *An. gambiae* | - | - | [39] |
| Tanzania | - | -999.00 | -99.00 | -8 | 1952 | -8 | 1952 | 94.0 | -8.0 | -8.0 | L | *An. gambiae* | - | - | [39] |
| Tanzania | - | -999.00 | -99.00 | -8 | 1953 | -8 | 1953 | 91.0 | -8.0 | -8.0 | P | *An. gambiae* | - | - | [39] |
| Tanzania | - | -999.00 | -99.00 | -8 | 1953 | -8 | 1953 | 90.0 | -8.0 | -8.0 | L | *An. gambiae* | - | - | [39] |
| Tanzania | Namawala | 36.40 | -8.15[r] | 02 | 1991 | 03 | 1991 | 82.7 | -8.0 | -8.0 | I | *An. arabiensis* | D | - | [3] |
| Tanzania | Namawala | 36.40 | -8.15[r] | -8 | -8 | -8 | -8 | 81.3 | -8.0 | -8.0 | M | *An. gambiae* s.l. | R | - | [40] |
| Tanzania | Namawala | 36.40 | -8.15[r] | 04 | 1991 | 05 | 1991 | 83.9 | -8.0 | -8.0 | P | *An. gambiae* s.l. | R | - | [40] |
| Tanzania | Namawala | 36.40 | -8.15[r] | -8 | -8 | -8 | -8 | -8.0 | 64.5 | 73.0 | M | *An. gambiae* s.l. | R | - | [40] |
| Tanzania | Namawala | 36.40 | -8.15[r] | -8 | 1990 | -8 | 1992 | 81.3 | -8.0 | -8.0 | P | *An. gambiae* s.l. | R | - | [40] |
| Tanzania | Michenga | 36.65 | -8.12[r] | -8 | 1989 | -8 | 1991 | 77.0 | -8.0 | -8.0 | P | *An. gambiae* s.l. | R | - | [40] |
| Tanzania | foothills of the Eastern Usambara Mountains | -999.00 | -99.00 | 03 | 1956 | 06 | 1959 | 84.1 | -8.0 | -8.0 | M | *An. gambiae* | - | - | [41] |
| Tanzania | Muheza | -999.00 | -99.00 | 11 | 1962 | 01 | 1964 | 85.4 | -8.0 | -8.0 | M | *An. gambiae* | - | - | [38] |
| Tanzania | Muheza | -999.00 | -99.00 | 11 | 1962 | 01 | 1964 | 85.0 | -8.0 | -8.0 | M | *An. funestus* | - | - | [38] |
| Tanzania | Gonja | -999.00 | -99.00 | 11 | 1962 | 01 | 1964 | 79.1 | -8.0 | -8.0 | M | *An. gambiae* | - | - | [38] |
| Tanzania | Ifakara | -999.00 | -99.00 | -9 | -9 | -9 | -9 | 78.0 | -9.0 | -9.0 | M | *An. gambiae* | - | - | [42] |
| Tanzania | 40 miles west of Tanga | -999.00 | -99.00 | 09 | 1952 | 12 | 1952 | -8.0 | 88.0 | 93.0 | S | *An. funestus* | R | - | [43] |
| Tanzania | 40 miles west of Tanga | -999.00 | -99.00 | 09 | 1952 | 12 | 1952 | -8.0 | 89.0 | 93.0 | S | *An. gambiae* | R | - | [43] |
| Tanzania | 40 miles west of Tanga | -999.00 | -99.00 | 09 | 1952 | 12 | 1952 | 93.7 | 83.0 | 98.5 | K | *An. funestus* | R | kept in cages | [43] |
| Tanzania | 40 miles west of Tanga | -999.00 | -99.00 | 09 | 1952 | 12 | 1952 | 96.3 | 92.0 | 99.1 | K | *An. gambiae* | R | kept in cages | [43] |
| Tanzania | coastal region | -999.00 | -99.00 | -8 | -8 | -8 | -8 | -8.0 | 92.0 | 93.0 | A | *An. gambiae* | - | - | [1] |
| Tanzania | South Pare district | -999.00 | -99.00 | -8 | -8 | -8 | -8 | -8.0 | 85.0 | 87.0 | A | *An. gambiae* | - | - | [1] |
| Tanzania | Muheza area, 5 villages | -999.00 | -99.00 | -8 | -8 | -8 | -8 | 81.9 | -8.0 | -8.0 | A | *An. gambiae* | - | - | [44] |
| Thailand | Ban Phluang | -999.00 | -99.00 | 06 | 1983 | 05 | 1984 | 89.0 | -8.0 | -8.0 | P | *An. dirus* | D | - | [45],b |
| Thailand | Ban Phluang | -999.00 | -99.00 | 06 | 1984 | 05 | 1985 | 83.0 | -8.0 | -8.0 | P | *An. dirus* | D | - | [45],b |
| Thailand | Ban Phluang | -999.00 | -99.00 | 06 | 1984 | 05 | 1985 | -8.0 | 59.0 | 84.0 | P | *An. dirus* | R/T | - | [45] |

to be continued

**Table 1 – continued**

| country | place | long [°E] | lat [°N] | M1 | YYY1 | M2 | YYY2 | $p_{d,ave}$ | $p_{d,min}$ | $p_{d,max}$ | way | species | wea | notes | ref |
|---|---|---|---|---|---|---|---|---|---|---|---|---|---|---|---|
| Uganda | - | -999.00 | -99.00 | -8 | -8 | -8 | -8 | 93.0 | -8.0 | -8.0 | P | *An. gambiae* | - | - | [39] |
| Uganda | - | -999.00 | -99.00 | -8 | -8 | -8 | -8 | 95.0 | -8.0 | -8.0 | L | *An. gambiae* | - | - | [39] |
| Uganda | - | -999.00 | -99.00 | -8 | -8 | -8 | -8 | 97.0 | -8.0 | -8.0 | S | *An. gambiae* | - | - | [39] |
| Uganda | Lira | -999.00 | -99.00 | 09 | 1953 | 12 | 1953 | 95.0 | -8.0 | -8.0 | P | *An. gambiae* | R | - | [46] |
| Uganda | Lira | -999.00 | -99.00 | 09 | 1953 | 12 | 1953 | 94.0 | -8.0 | -8.0 | P | *An. funestus* | R | - | [46] |
| Uganda | Lira | -999.00 | -99.00 | 09 | 1953 | 12 | 1953 | 94.5 | -8.0 | -8.0 | K | *An. gambiae* | R | - | [46] |
| Uganda | Lira | -999.00 | -99.00 | 09 | 1953 | 12 | 1953 | 94.6 | -8.0 | -8.0 | K | *An. funestus* | R | - | [46] |
| Uganda | Lira | -999.00 | -99.00 | 09 | 1953 | 12 | 1953 | 97.0 | 94.0 | 99.0 | S | *An. gambiae* | R | - | [46] |
| Uganda | Lira | -999.00 | -99.00 | 09 | 1953 | 12 | 1953 | 93.0 | 88.0 | 94.0 | S | *An. gambiae* | R | - | [46] |
| USA | Sheridan | -999.00 | -99.00 | 08 | 1984 | 09 | 1984 | 72.0 | -9.0 | -9.0 | -9 | *An. freeborni* | - | unfed | [47] |
| USA | Sheridan | -999.00 | -99.00 | 08 | 1984 | 09 | 1984 | 74.0 | -9.0 | -9.0 | -9 | *An. freeborni* | - | blood-fed | [47] |
| USA | Sheridan | -999.00 | -99.00 | 08 | 1984 | 09 | 1984 | 75.0 | -9.0 | -9.0 | K | *An. freeborni* | - | - | [47] |

## References


1. Gillies MT: **A modified technique for the age-grading of populations of *Anopheles gambiae***. *Ann Trop Med Parasitol* 1958, **52**:261–273.
2. Draper CC, Davidson G: **A new method of estimating the survival-rate of anopheline mosquitoes in nature.** *Nature* 1953, **172**:503.
3. Charlwood JD, Kihonda J, Sama S, Billingsley PF, Hadji H, Verhave JP, Lyimo E, Luttikhuizen PC, Smith T: **The rise and fall of *Anopheles arabiensis* (Diptera: Culicidae) in a Tanzanian village.** *Bull Entomol Res* 1995, **85**:37–44.
4. Hay SI, Guerra CA, Tatem AJ, Atkinson PM, Snow RW: **Urbanization, malaria transmission and disease burden in Africa.** *Nat Rev Microbiol* 2005, **3**:81–90.
5. Hay SI, Rogers DJ, Toomer JF, Snow RW: **Annual *Plasmodium falciparum* entomological inoculation rates (EIR) across Africa: literature survey, internet access and review.** *Trans R Soc Trop Med Hyg* 2000, **94**:113–127.
6. Dos Santos RlC, Forattini OP, Burattini MN: ***Anopheles albitarsis s.l.* (Diptera Culicidae) survivorship and density in a rice irrigation area of the state of São Paulo, Brazil.** *J Med Entomol* 2004, **41**:997–1000.
7. Garrett-Jones C, Grab B: **The assessment of insecticidal impact on the malaria mosquito's vectorial capacity, from data on the proportion of parous females.** *Bull World Health Org* 1964, **31**:71–86.
8. Robert V, Gazin P, Benasseni R, Carnevale P: **Le paludisme urbain à Bobo-Dioulasso (Burkina Faso).** In *Urbanisation et santé dans le Tiers Monde: transition épidémiologique, changement social et soins de santé primaires*. Edited by Salem G, Emile J, Paris, France: ORSTROM 1989:181–185.
9. Costantini C, Song-Gang L, della Torre A, Sagnon N, Coluzzi M, Taylor CE: **Density, survival and dispersal of *Anopheles gambiae* complex mosquitoes in a West African Sudan savanna village.** *Med Vet Entomol* 1996, **10**:203–219.
10. Robert V, van den Broek A, Stevens P, Slootweg R, Petrarca V, Coluzzi M, Le Goff G, Deco MAD, Carnevale P: **Mosquitoes and malaria transmission in irrigated rice-fields in the Benoue Valley of Northern Cameroon.** *Acta Trop* 1992, **52**:201–204.
11. Dossou-Yovo J, Doannio JMC, Diarrassouba S, Chauvancy G: **Malaria in Côte d'Ivoire wet savannah region: the entomological input.** *Trop Med Parasitol* 1995, **46**:263–269.
12. Mekuria YR, Granados R, Tidwell MA, Williams DC, Wirtz RA, Roberts DR: **Malaria transmission potential by *Anopheles* mosquitoes of Dajabon, Dominican Republic.** *J Am Mosq Control Assoc* 1991, **7**:456–461.
13. Kenawy MA: **Development and survival of *Anopheles pharoensis* and *An. multicolor* from Faiyum, Egypt.** *J Am Mosq Control Assoc* 1991, **7**:551–555.
14. Beier MS, Beier JC, Merdan AA, Sawaf BME, Kadder MA: **Laboratory rearing techniques and adult life table parameters for *Anopheles sergentii* from Egypt.** *J Am Mosq Control Asso* 1987, **3**:266–270.

15. Weidhaas DE, Breeland SG, Lofgren CS, Dame DA, Kaiser R: **Release of chemosterilized males for the control of *Anopheles Albimanus* in El Salvador. IV. Dynamics of the test population.** *Am J Trop Med Hyg* 1974, **23**:298–308.
16. Zaim M, Zahirnia AH, Manouchehri AV: **Survival rates of *Anopheles culicifacies s.l.* and *Anopheles pulcherrimus* in sprayed and unsprayed villages in Ghassreghand district, Baluchistan, Iran, 1991.** *J Am Mosq Control Asso* 1993, **9**:421–425.
17. McDonald PT: **Population characteristics of domestic *Aedes aegypti* (Diptera: Culicidae) in villages on the Kenya coast. I. Adult survivorship and population size.** *J Med Entomol* 1977, **14**:42–48.
18. Mutero CM, Birley MH: **Estimation of the survival rate and oviposition cycle of field populations of malaria vectors in Kenya.** *J Appl Ecol* 1987, **24**:853–863.
19. Lee HI, Lee JS, Shin EH, Lee WJ, Kim YY, Lee KR: **Malaria transmission potential by *Anopheles sinensis* in the Republic of Korea.** *Korean J Parasitol* 2001, **39**:185–192.
20. Ree HI, Hwang UW: **Comparative study on longevity of *Anopheles sinensis* in malarious and non-malarious areas in Korea.** *Korean J Parasitol* 2000, **38**:263–266.
21. Ree HI, Hwang UW, Lee IY, Kim TE: **Daily survival and human blood index of *Anopheles sinensis*, the vector species of malaria in Korea.** *J Am Mosq Control Asso* 2001, **17**:67–72.
22. Shin EH, Lee WJ, Lee HI, Lee DK, Klein TA: **Seasonal population density and daily survival of anopheline mosquitoes (Diptera: Culicidae) in a malaria endemic area, Republic of Korea.** *J Vector Ecol* 2005, **30**:33–40.
23. Arredondo-Jimenez JI, Rodriguez MH, Washino RK: **Gonotrophic cycle and survivorship of *Anopheles vestitipennis* (Diptera: Culicidae) in two different ecological areas of southern Mexico.** *J Med Entomol* 1998, **35**:937–942.
24. Fernandez-Salas I, Rodriguez MH, Roberts DR: **Gonotrophic cycle and survivorship of *Anopheles pseudopunctipennis* (Diptera: Culicidae) in the Tapachula foothills of southern Mexico.** *J Med Entomol* 1994, **31**:340–347.
25. Garrett-Jones C, Shidrawi GR: **Malaria vectorial capacity of a population of *Anopheles gambiae*: an exercise in epidemiological entomology.** *Bull World Health Org* 1969, **40**:531–545.
26. Service MW: **Some basic entomological factors concerned with the transmission and control of malaria in northern Nigeria.** *Trans R Soc Trop Med Hyg* 1965, **59**:292–296.
27. Khan AQ, Talibi SA: **Epidemiological assessment of malaria transmission in an endemic area of East Pakistan and the significance of congenital immunity.** *Bull World Health Org* 1972, **46**:783–792.
28. Reisen WK, Aslamkhan M: **A release-recapture experiment with the malaria vector, *Anopheles stephensi Liston*, with observations on dispersal, survivorship, population size, gonotrophic rhythm and mating behaviour.** *Ann Trop Med Parasitol* 1979, **73**:251–269.
29. Graves PM, Burkot TR, Saul AJ, Hayes RJ, Carter R: **Estimation of anopheline survival rate, vectorial capacity and mosquito infection probability from malaria vector infection rates in villages near Madang, Papua New Guinea.** *J Appl Ecol* 1990, **27**:134–147.
30. Killeen GF, McKenzie FE, Foy BD, Schieffelin C, Billingsley PF, Beier JC: **A simplified model for predicting malaria entomologic inoculation rates based on entomologic and parasitologic parameters relevant to control.** *Am J Trop Med Hyg* 2000, **62**:535–544.
31. Vercruysse J: **Étude entomologique sur la transmission du paludisme humain dans le bassin du fleuve Sénégal (Senegal).** *Ann Soc Belg Med Trop* 1985, **65 (Suppl. 2)**:171–179.
32. Ndiaye PI, Bicout DJ, Mondet B, Sabatier P: **Rainfall triggered dynamics of *Aedes* mosquito aggressiveness.** *J Theor Biol* 2006, **243**:222–229.
33. Vercruysse J, Jancloes M, van de Velden L: **Epidemiology of seasonal falciparum malaria in an urban area of Senegal.** *Bull World Health Org* 1983, **61**:821–831.
34. Vercruysse J: **Estimation of the survival rate of *Anopheles arabiensis* in an urban area (Pikine-Senegal).** *J Anim Ecol* 1985, **54**:343–350.

35. Bockarie MJ, Service MW, Barnish G, Touré YT: **Vectorial capacity and entomological inoculation rates of *Anopheles gambiae* in a high rainfall forested area of southern Sierra Leone.** *Trop Med Parasitol* 1995, **46**:164–171.
36. De Zoysa APK, Herath PRJ, Abhayawardana TA, Padmalal UKGK, Mendis KN: **Modulation of human malaria transmission by anti-gamete transmission blocking immunity.** *Trans R Soc Trop Med Hyg* 1988, **82**:548–553.
37. El Sayed BB, Arnot DE, Mukhtar MM, Baraka OZ, Dafalla AA, Elnaiem DEA, Nugud AHD: **A study of the urban malaria transmission problem in Khartoum.** *Acta Trop* 2000, **75**:163–171.
38. Gillies MT, Wilkes TJ: **A study on the age-composition of populations of *Anopheles gambiae* Giles and *A. funestus* Giles in north-eastern Tanzania.** *Bull Entomol Res* 1965, **56**:237–262.
39. Davidson G: **Estimation of the survival-rate of anopheline mosquitoes in nature.** *Nature* 1954, **174**:792–793.
40. Charlwood JD, Smith T, Billingsley PF, Takken W, Lyimo EOK, Meuwissen JHET: **Survival and infection probabilities of anthropophagic *anophelines* from an area of high prevalence of *Plasmodium falciparum* in humans.** *Bull Entomol Res* 1997, **87**:445–453.
41. Gillies MT: **Studies on the dispersion and survival of *Anopheles gambiae* Giles in East Africa, by means of marking and release experiments.** *Bull Entomol Res* 1961, **52**:99–127.
42. Takken W, Charlwood JD, Billingsley PF, Gort G: **Dispersal and survival of *Anopheles funestus* and *A. gambiae s. l.* (Diptera: Culicidae) during the rainy season in Southeast Tanzania.** *Bull Entomol Res* 1998, **88**:561–566.
43. Davidson G, Draper CC: **Field studies of some of the basic factors concerned in the transmission of malaria.** *Trans R Soc Trop Med Hyg* 1953, **47**:522–535.
44. Lines JD, Wilkes TJ, Lyimo EO: **Human malaria infectiousness measured by age-specific sporozoite rates in *Anopheles gambiae* in Tanzania.** *Parasitology* 1991, **102 (Suppl.)**:167–177.
45. Rosenberg R, Andre RG, Somchit L: **Highly efficient dry season transmission of malaria in Thailand.** *Trans R Soc Trop Med Hyg* 1990, **84**:22–28.
46. Davidson G: **Further studies of the basic factors concerned in the transmission of malaria.** *Trans R Soc Trop Med Hyg* 1955, **49**:339–350.
47. McHugh CP: **Ecology of a semi-isolated population of adult *Anopheles freeborni*: abundance, trophic status, parity, survivorship, gonotrophic cycle length, and host selection.** *Am J Trop Med Hyg* 1989, **41**:169–176.
