# Supplementary material for: Development of a new version of the Liverpool Malaria Model. I. Refining the parameter settings and mathematical formulation of basic processes based on a literature review
Source: Malar J. 2011 Feb 11;10:35. doi: 10.1186/1475-2875-10-35 (PMC3055220; doi:10.1186/1475-2875-10-35)
Supplement: Additional file 5 — Mosquito-to-human transmission efficiencies. Data regarding the mosquito-to-human transmission efficiency (b). [file 1475-2875-10-35-S5.PDF]

5 Mosquito-to-human transmission efficiencies

Data regarding the mosquito-to-human transmission efficiency (b).

Columns: country: country where the study was undertaken; place: location of the study site; long: longitude of the study site (-999.00: position is either unknown or was not seeked out); lat: latitude of the study site (-99.00: position is either unknown or was not seeked out); M1: month, when the study started; YYY1: year of the start of the study; M2: month, when the study ended; YYY2: year of the end of the study; b_{ave}: average mosquito-to-human transmission efficiency; b_{min}: as b_{ave}, but for the minimum; b_{max}: as b_{ave}, but for the maximum; species: involved mosquito species; notes: notes; ref: reference. The '-8' denotes data that were not available in the literature and that could not be checked due to limited access, respectively. Note that minimum and maximum values of b refer to monthly data. Indices: ^{b}: the position of the study site was extracted from Hay *et al.* [1]. Symbols: ⋆: children (the following numbers indicate age classes in years); ★: adults; ⧫: all ages.

country	place	long [°E]	lat [°N]	M1	YYY1	M2	YYY2	b_{ave} [%]	b_{min} [%]	b_{max} [%]	species	notes	ref
-	-	-999.00	-99.00	-8	-8	-8	-8	44.1	-8.0	-8.0	*An. gambiae*	in vitro transmitted sporozoites	[2]
-	-	-999.00	-99.00	-8	-8	-8	-8	49.2	-8.0	-8.0	*An. stephensi*	in vitro transmitted sporozoites	[2]
Kenya	Nyanza Province	-999.00	-99.00	08	1972	07	1973	1.5-2.6	-8.0	-8.0	*An. gambiae*, *An. funestus*	infants; neglected HBR age-dependence; ignored superinfection	[3]
Kenya	Nyanza Province	-999.00	-99.00	08	1972	07	1973	5.4-9.3	-8.0	-8.0	*An. gambiae*, *An. funestus*	infants; ignored superinfection	[4]
-	-	-999.00	-99.00	-8	-8	-8	-8	33.0	-8.0	-8.0	*An. stephensi*	★(25-39); without antimalarial immunity	[5]
Kenya	Saradidi	-999.00	-99.00	02	1986	10	1987	7.5	1.0	28.0	*An. gambiae*, *An. arabiensis*, *An. funestus*	⋆(0.5-6); neglected HBR age-dependence; ignored superinfection	[6]
Uganda	Lira	-999.00	-99.00	-8	-8	-8	-8	5.0	-8.0	-8.0	*An. gambiae*, *An. funestus*	infants; neglected HBR age-dependence; ignored superinfection	[7,8]
Tanzania	Mngeza	-999.00	-99.00	-8	-8	-8	-8	1.0	-8.0	-8.0	*An. gambiae*, *An. funestus*	infants; neglected HBR age-dependence; ignored superinfection	[7,8]
Ethiopia	Gambela	-999.00	-99.00	12	1967	02	1969	-8.0	6.6	27.3	*An. arabiensis*, *An. funestus*, *An. nili*	⋆($<$15); neglected HBR age-dependence; ignored superinfection; assumption: $r = 0.005$	[9]
Ethiopia	Gambela	-999.00	-99.00	12	1967	02	1969	-8.0	4.8	13.3	*An. arabiensis*, *An. funestus*, *An. nili*	★($\geq$15); neglected HBR age-dependence; ignored superinfection; assumption: $r = 0.005$	[9]
Senegal	Pikine	-17.40	14.75^{b}	01	1980	01	1981	-8.0	8.0	46.1	*An. arabiensis*	⋆(0.5-6); ignored superinfection	[10]

References

1. Hay SI, Rogers DJ, Toomer JF, Snow RW: **Annual *Plasmodium falciparum* entomological inoculation rates (EIR) across Africa: literature survey, internet access and review.** *Trans R Soc Trop Med Hyg* 2000, **94**:113–127.
2. Beier JC, Davis JR, Vaughan JA, Noden BH, Beier MS: **Quantitation of *Plasmodium falciparum* sporozoites transmitted in vitro by experimentally infected *Anopheles gambiae* and *Anopheles stephensi*.** *Am J Trop Med Hyg* 1991, **44**:564–570.
3. Pull JH, Grab B: **A simple epidemiological model for evaluating the malaria inoculation rate and the risk of infection in infants.** *Bull World Health Org* 1974, **51**:507–516.
4. Port GR, Boreham PFL, Bryan JH: **The relationship of host size to feeding by mosquitoes of the *Anopheles gambiae* Giles complex (Diptera: Culicidae).** *Bull Entomol Res* 1980, **70**:133–144.
5. Rickman L, Jones TR, Long GW, Paparello S, Schneider I, Paul CF, Beaudoin RL, Hoffman SL: ***Plasmodium falciparum*-infected *Anopheles stephensi* inconsistently transmit malaria to humans.** *Am J Trop Med Hyg* 1990, **43**:441–445.
6. Beier JC, Oster CN, Onyango FK, Bales JD, Sherwood JA, Perkins PV, Chumo DK, Koech DV, Whitmire RE, Roberts CR, Diggs CL, Hoffman SL: ***Plasmodium falciparum* incidence relative to entomologic inoculation rates at a site proposed for testing malaria vaccines in western Kenya.** *Am J Trop Med Hyg* 1994, **50**:529–536.
7. Macdonald G: **The measurement of malaria transmission.** *Proc R Soc Med* 1955, **48**:295–301.
8. Macdonald G: **Epidemiological basis of malaria control.** *Bull World Health Org* 1956, **15**:613–626.
9. Krafsur ES, Armstrong JC: **An integrated view of entomological and parasitological observations on falciparum malaria in Gambela, Western Ethiopian Lowlands.** *Trans R Soc Trop Med Hyg* 1978, **72**:348–356.
10. Vercruysse J, Jancloes M, van de Velden L: **Epidemiology of seasonal falciparum malaria in an urban area of Senegal.** *Bull World Health Org* 1983, **61**:821–831.
