# Supplementary material for: Development of a new version of the Liverpool Malaria Model. I. Refining the parameter settings and mathematical formulation of basic processes based on a literature review
Source: Malar J. 2011 Feb 11;10:35. doi: 10.1186/1475-2875-10-35 (PMC3055220; doi:10.1186/1475-2875-10-35)
Supplement: Additional file 6 — Sexual parasite ratios. Data with regard to the sexual parasite ratio (sPR), that is the percentage of humans with gametocytes in their blood as well as the ratio between sexual and asexual parasite ratio (SAR), which is the proportion of malaria parasite positive humans that are gametocytaemic. [file 1475-2875-10-35-S6.PDF]

6 Sexual parasite ratios

Data with regard to the sexual parasite ratio (*sPR*), that is the percentage of humans with gametocytes in their blood as well as the ratio between sexual and asexual parasite ratios (*SAR*), which is the proportion of malaria parasite positive humans that are gametocytaemic. Note that particular values of the asexual parasite ratio (*PR*) can be calculated via $PR = \frac{sPR}{SAR}$.

Columns: country: country where the study was undertaken; place: location of the study site; long: longitude of the study site (-999.00: position is either unknown or was not seeked out); lat: latitude of the study site (-99.00: position is either unknown or was not seeked out); M1: month, when the study started; YYY1: year of the start of the study; M2: month, when the study ended; YYY2: year of the end of the study; sPR_a: annual mean *sPR*; $sPR_{min,a}$: annual minimum *sPR*; $sPR_{max,a}$: annual maximum *sPR*; SAR_a: annual mean *SAR*; $SAR_{min,a}$: annual minimum *SAR*; $SAR_{max,a}$: annual maximum *SAR*; U_b: land use classification as defined by Hay *et al.* [1]: R=rural, I=irrigated rice, U= urban area; U_a: as U_b but after Hay *et al.* [2]: PU=peri-urban (population densities of 250-1,000 persons per km^2), R1=rural 1 (population densities of 100-250 persons per km^2), R2=rural 2 (population densities of <100 persons per km^2), U=urban (population densities of $>1{,}000$ persons per km^2); U_p: as U_a, but as taken from the particular reference: R=rural area, I=irrigation/permanent stream or river, U=urban area; notes: notes; ref: reference. The '-8' and '-9' denote data that were not available in the literature and that could not be checked due to limited access, respectively. Indices: [a]: the position of the study site was taken from Hay *et al.* [2]; [b]: the position of the study site was extracted from Hay *et al.* [1]; [i]: the position of the study site was taken from http://www.heavens-above.com/countries.aspx; [m]: the position of the study site was derived from a published map; [r]: the position of the study site was found in the reference. Symbols: ⋆: children (the following numbers indicate age classes in years); ★: adults; ⧫: all ages.

country	place	long [°E]	lat [°N]	M1	YYY1	M2	YYY2	sPR_a	$sPR_{min,a}$	$sPR_{max,a}$	SAR_a	$SAR_{min,a}$	$SAR_{max,a}$	U_b [1]	U_a [2]	U_p	notes	ref
Burkina Faso	Bobo-Dioulasso, Colma-Nord quarter	-4.30	11.21[b]	01	1985	12	1985	10.5	5.6	21.1	36.1	26.7	44.3	U/R	R1	R	⋆(0-15)	[3]
Burkina Faso	Bobo-Dioulasso, Diaradougou quarter	-4.29	11.18[b]	01	1985	12	1985	2.3	0.6	4.7	33.3	12.5	45.5	U	R1	U	⋆(0-15)	[3]
Burkina Faso	Bobo-Dioulasso, Dioulassoba quarter	-4.30	11.19[b]	01	1985	12	1985	3.1	2.6	3.4	28.0	19.0	57.1	U	R1	U	⋆(0-15)	[3]
Burkina Faso	Bouloy, Kolel, Bella and Peul Djelgobé camps	-999.00	-99.00	06	1985	03	1986	1.7	0.0	5.0	12.9	0.0	16.7	-8	-8	R	★	[4]
Burkina Faso	Bouloy, Kolel, Bella and Peul Djelgobé camps	-999.00	-99.00	06	1985	03	1986	8.6	7.5	9.1	17.0	12.0	24.1	-8	-8	R	⋆(0.5-15)	[4]
Burkina Faso	Karangasso	-4.63	11.22[b]	02	1985	02	1986	21.3	4.5	30.0	38.3	6.0	52.7	R	R2	R	⋆(5-9)	[5,6]
Burkina Faso	Karangasso	-4.63	11.22[b]	02	1985	02	1986	25.5	9.2	37.3	40.8	24.2	63.9	R	R2	R	⋆(0-4)	[5,6]
Burkina Faso	Karangasso	-4.63	11.22[b]	02	1985	02	1986	29.4	8.5	39.7	47.8	11.8	65.1	R	R2	R	⋆(10-14)	[5,6]
Burkina Faso	near Bobo-Dioulasso	-999.00	-99.00	-8	1985	-8	1987	10.9	-8.0	-8.0	-8.0	-8.0	-8.0	R	R2	R	⧫	[7]
Burkina Faso	Ouagadougou	-999.00	-99.00	08	1984	09	1984	-8.0	-8.0	7.6	25.9	-8.0	-8.0	-8	-8	U	⋆(0-5); more detailed information is available	[8]
Burkina Faso	Oursi and Déou	-999.00	-99.00	06	1985	03	1986	4.4	0.0	15.2	16.5	0.0	33.3	-8	-8	R	⋆(0.5-15)	[4]
Burkina Faso	6 villages north of Ouagadougou	-999.00	-99.00	12	2003	12	2003	-8.0	21.4	-8.0	-8.0	28.5	-8.0	-8	-8	R	⧫; microscope detection	[9]

to be continued

Table 1 – continued

country	place	long [°E]	lat [°N]	M1	YYY1	M2	YYY2	sPR_a	$sPR_{min,a}$	$sPR_{max,a}$	SAR_a	$SAR_{min,a}$	$SAR_{max,a}$	U_b [1]	U_a [2]	U_p	notes	ref
Burkina Faso	6 villages north of Ouagadougou	-999.00	-99.00	12	2003	12	2003	-8.0	70.1	-8.0	-8.0	73.6	-8.0	-8	-8	R	◆; QT-NASBA detection	[9]
Burkina Faso	Tin Edjar	-0.68	14.69[m]	06	1985	03	1986	0.0	0.0	0.0	0.0	0.0	0.0	-8	-8	R	★	[4]
Burkina Faso	Tin Edjar	-0.68	14.69[m]	06	1985	03	1986	4.2	2.6	5.4	12.5	8.0	26.7	-8	-8	R	⋆(0.5-15)	[4]
Burkina Faso	VK4	-4.42	11.37[b]	01	1985	02	1986	12.0	3.2	16.2	37.2	18.0	56.7	I	R2	I	⋆(0-4)	[6]
Burkina Faso	VK4	-4.42	11.37[b]	01	1985	02	1986	13.9	7.2	23.8	32.2	22.6	48.4	I	R2	I	⋆(5-9)	[6]
Burkina Faso	VK4	-4.42	11.37[b]	01	1985	02	1986	6.2	0.0	19.1	17.0	0.0	56.2	I	R2	I	⋆(10-14)	[6]
Cameroon	Bondi	12.19	3.86[m]	-8	1998	-8	2000	11.8	8.7	15.0	23.7	17.2	30.3	-8	-8	R	◆(0.8-77); area: degraded forest; position derived from [10]	[11]
Cameroon	Ebolakounou	12.13	3.93[a]	05	1996	05	1998	4.7	-8.0	-8.0	13.7	-8.0	-8.0	-8	R2	R	★(>15); forest area	[12]
Cameroon	Ebolakounou	12.13	3.93[a]	05	1996	05	1998	9.1	-8.0	-8.0	14.7	-8.0	-8.0	-8	R2	R	⋆(0-15); forest area	[12]
Cameroon	Koundou	12.12	3.90[a]	05	1996	05	1998	10.3	-8.0	-8.0	14.8	-8.0	-8.0	-8	R2	R	⋆(0-15); degraded forest	[12]
Cameroon	Koundou	12.12	3.90[a]	05	1996	05	1998	3.8	-8.0	-8.0	9.6	-8.0	-8.0	-8	R2	R	★(>15); degraded forest	[12]
Cameroon	Mengang	12.05	3.88[i]	-8	1998	-8	2000	23.6	12.9	34.3	33.7	22.1	42.0	-8	-8	R	◆(0.8-77); area: degraded forest	[11]
Cameroon	Mengang and Yaounde	-999.00	-99.00	-8	-8	-8	-8	10.4	-8.0	-8.0	15.6	-8.0	-8.0	-8	-8	R&U	⋆(0-14)	[13]
Cameroon	Mengang and Yaounde	-999.00	-99.00	-8	-8	-8	-8	4.8	-8.0	-8.0	11.5	-8.0	-8.0	-8	-8	R&U	★(>14)	[13]
Cameroon	Mengang district, 2 villages	-999.00	-99.00	-8	-8	-8	-8	15.7	-8.0	-8.0	27.7	-8.0	-8.0	-8	-8	R	-	[14]
Cameroon	Yaoundé, Messa quarter	-999.00	-99.00	10	1990	01	1993	5.4	-8.0	-8.0	14.6	-8.0	-8.0	-8	-8	U	◆(4-60)	[15]
Cameroon	Yaoundé and Mengang	-999.00	-99.00	-8	-8	-8	-8	19.1	-8.0	-8.0	40.2	-8.0	-8.0	-8	-8	-8	detected by quantitative buffy coat tests; patients presenting clinical malaria and ⋆(3-15)	[16]
Cameroon	Yaoundé and Mengang	-999.00	-99.00	-8	-8	-8	-8	20.0	-8.0	-8.0	39.8	-8.0	-8.0	-8	-8	-8	detected by thick blood films and quantitative buffy coat tests; patients presenting clinical malaria and ⋆(3-15)	[16]
Cameroon	Yaoundé and Mengang	-999.00	-99.00	-8	-8	-8	-8	4.6	-8.0	-8.0	10.1	-8.0	-8.0	-8	-8	-8	detected by thick blood films; patients presenting clinical malaria and ⋆(3-15)	[16]
Cameroon	Yaoundé, Dakar quarter	11.52	3.87[i]	07	1999	05	2000	4.3	0.0	7.0	12.6	0.0	21.9	-8	-8	U	◆; microscope; drug influenced	[17]
Cameroon	Yaoundé, Essos	11.00	3.00[b]	06	1989	02	1990	3.2	2.4	7.4	8.5	0.0	16.7	-8	-8	U	⋆(0-15)	[18]
Cameroon	Yaoundé, Obili district	11.52	3.87[i]	10	1989	07	1990	1.1	0.0	3.0	4.0	0.0	7.7	-8	U	U	⋆(0-15)	[18]
Côte d'Ivoire	Katiola district, 8 villages (no rice cultivation)	-999.00	-99.00	03	1997	01	1998	13.0	11.0	14.0	14.9	13.3	15.6	-8	-8	R	⋆(0-9); villages are Angolokaha, Doussoulokaha, Folofonkaha, Kabolo, Ounadiékaha, Petionara, Sérigobokaha, and Timorokaha	[19]
Côte d'Ivoire	Katiola district, 8 villages (no rice cultivation)	-999.00	-99.00	03	1997	01	1998	4.0	3.0	5.0	6.5	5.4	7.6	-8	-8	R	PR: ⋆(≥10); see above	[19]
Côte d'Ivoire	Korhogo district, 8 villages (rice cultivation during the dry season)	-999.00	-99.00	03	1997	01	1998	11.0	10.0	11.0	13.9	13.3	13.6	-8	-8	I	⋆(0-9); villages are Gbahaouakaha, Kohotieri, Koumbolikaha, Lamékaha, Nambêkaha, Nombolo, Nongotchénékaha, and Zémongokaha	[19]
Côte d'Ivoire	Korhogo district, 8 villages (rice cultivation during the dry season)	-999.00	-99.00	03	1997	01	1998	4.0	4.0	5.0	9.2	10.0	10.3	-8	-8	I	subjects (≥10); see above	[19]
Côte d'Ivoire	Korhogo district, 8 villages (rice cultivation during the rainy season)	-999.00	-99.00	03	1997	01	1998	12.0	12.0	12.0	14.3	14.0	15.0	-8	-8	R	⋆(0-9); villages are Binguebougou, Fapaha, Kombolokoura, Kaforo, Karakpo, Kassoumbarga, Katiorkpo, and Tioro	[19]
Côte d'Ivoire	Korhogo district, 8 villages (rice cultivation during the rainy season)	-999.00	-99.00	03	1997	01	1998	4.0	3.0	5.0	6.1	4.9	7.2	-8	-8	R	⋆(≥10); see above	[19]
Congo	Linzolo	15.11	-4.41[b]	11	1981	05	1985	22.5	21.8	27.7	28.5	27.6	34.2	R	R1	R	⋆(0-14); microscope	[20]
Congo	6 villages near Brazzaville	-999.00	-99.00	11	1981	05	1985	28.4	-8.0	-8.0	34.0	-8.0	-8.0	-8	-8	R	⋆(5-15) (from school); microscope	[20]
Gambia	4 villages west of Farafenni	-999.00	-99.00	05	2001	06	2001	-8.0	13.5	-8.0	-8.0	24.9	-8.0	-8	-8	R	⋆(0.5-15)	[21]
Gambia, The	5 villages around Farafenni	-16.57	13.47[r]	04	2003	05	2003	-8.0	-8.0	3.1	-8.0	-8.0	15.3	-8	-8	R	◆(5-45); detected by microscopy	[22]
Gambia, The	5 villages around Farafenni	-16.57	13.47[r]	04	2003	05	2003	-8.0	-8.0	3.1	-8.0	-8.0	15.3	-8	-8	R	◆(5-45); detected by microscopy	[22]
Gambia, The	5 villages around Farafenni	-16.57	13.47[r]	05	2003	10	2003	24.2	-8.0	-8.0	44.9	-8.0	-8.0	-8	-8	R	◆(5-45); detected by microscopy	[22]
Gambia, The	5 villages around Farafenni	-16.57	13.47[r]	05	2003	10	2003	51.6	-8.0	-8.0	62.7	-8.0	-8.0	-8	-8	R	◆(5-45); PCR/RT-PCR detection	[22]
Gambia, The	near Farafenni, north bank villages	-999.00	-99.00	06/11	1990 1991	06/11	1990 1991	12.3	-8.0	-8.0	-8.0	-8.0	-8.0	-8	-8	-8	⋆(1-19)	[23]
Gambia, The	near Farafenni, north bank villages	-999.00	-99.00	06/11	1990 1991	06/11	1990 1991	6.1	-8.0	-8.0	-8.0	-8.0	-8.0	-8	-8	-8	★(>20)	[23]
Gambia, The	near Farafenni, north bank villages	-999.00	-99.00	-8	-8	-8	-8	-8.0	-8.0	-8.0	-8.0	11.7	31.0	-8	-8	-8	★(>14); graphically derived	[24]
Gambia, The	near Farafenni, north bank villages	-999.00	-99.00	-8	-8	-8	-8	-8.0	-8.0	-8.0	-8.0	18.8	43.5	-8	-8	-8	⋆(0-14); graphically derived	[24]
Gambia, The	near Farafenni, south bank villages	-999.00	-99.00	-8	-8	-8	-8	-8.0	-8.0	-8.0	-8.0	15.3	26.0	-8	-8	-8	★(>14); graphically derived	[24]
Gambia, The	near Farafenni, south bank villages	-999.00	-99.00	-8	-8	-8	-8	-8.0	-8.0	-8.0	-8.0	16.5	27.0	-8	-8	-8	⋆(0-14); graphically derived	[24]
Ghana	-9	-999.00	-99.00	01	1952	10	1952	20.0	-9.0	-9.0	-9.0	-9.0	-9.0	-8	-8	-9	⋆(0-14)	[25]
Ghana	-9	-999.00	-99.00	01	1952	10	1952	6.0	-9.0	-9.0	-9.0	-9.0	-9.0	-8	-8	-9	★(>14)	[25]
Ghana	Weija	-999.00	-99.00	01	1952	10	1952	20.0	-8.0	-8.0	22.9	-8.0	-8.0	-8	-8	R	⋆(0.8-10)	[25]
Kenya	Ahero	-999.00	-99.00	-8	1935	-8	1936	51.6	-8.0	-8.0	61.5	-8.0	-8.0	-8	-8	-8	⋆(0-4); microscope detection	[26]
Kenya	Chonyi	-999.00	-99.00	-8	-8	-8	-8	-8.0	-8.0	-8.0	17.3	-8.0	-8.0	-8	-8	-8	⋆(0-14); graphically derived	[24]
Kenya	Chonyi	-999.00	-99.00	-8	-8	-8	-8	-8.0	-8.0	-8.0	3.7	-8.0	-8.0	-8	-8	-8	★(>14); graphically derived	[24]
Kenya	Kanyamedha	-999.00	-99.00	-8	1935	-8	1936	24.2	-8.0	-8.0	29.1	-8.0	-8.0	-8	-8	-8	⋆(0-4); microscope detection	[26]
Kenya	Kasagam	-999.00	-99.00	-8	1935	-8	1936	9.3	-8.0	-8.0	10.7	-8.0	-8.0	-8	-8	-8	⋆(0-4); microscope detection	[26]
Kenya	Kisumu	-999.00	-99.00	-8	1935	-8	1936	17.9	-8.0	-8.0	25.2	-8.0	-8.0	-8	-8	-8	⋆(0-4); microscope detection	[26]
Kenya	Ngerenya	-999.00	-99.00	-8	-8	-8	-8	-8.0	-8.0	-8.0	23.8	-8.0	-8.0	-8	-8	-8	⋆(0-14); graphically derived	[24]
Kenya	Ngerenya	-999.00	-99.00	-8	-8	-8	-8	-8.0	-8.0	-8.0	7.3	-8.0	-8.0	-8	-8	-8	★(>14); graphically derived	[24]
Kenya	Nyakatch	-999.00	-99.00	-8	1935	-8	1936	37.7	-8.0	-8.0	49.1	-8.0	-8.0	-8	-8	-8	⋆(0-4); microscope detection	[26]
Liberia	-9	-999.00	-99.00	-9	-9	-9	-9	15.6	-9.0	-9.0	-9.0	-9.0	-9.0	-8	-8	-9	⋆(0-14)	[27]
Liberia	-9	-999.00	-99.00	-9	-9	-9	-9	5.5	-9.0	-9.0	-9.0	-9.0	-9.0	-8	-8	-9	★(>14)	[27]
Liberia	Marshall Territory	-999.00	-99.00	01	195?	12	195?	6.0	-8.0	-8.0	31.4	-8.0	-8.0	-8	-8	-8	-	[28]
Nigeria	Garki, village 154	-999.00	-99.00	-9	-9	-9	-9	36.5	-8.0	-8.0	-8.0	-8.0	-8.0	-8	-8	-9	⋆(1-8)	[29]

to be continued

Table 1 – continued

country	place	long [°E]	lat [°N]	M1	YYY1	M2	YYY2	sPR_a	$sPR_{min,a}$	$sPR_{max,a}$	SAR_a	$SAR_{min,a}$	$SAR_{max,a}$	U_b [1]	U_a [2]	U_p	notes	ref
Nigeria	Garki, village 154	-999.00	-99.00	-9	-9	-9	-9	9.8	-8.0	-8.0	-8.0	-8.0	-8.0	-8	-8	-9	◆(>9)	[29]
Nigeria	Garki, village 202	-999.00	-99.00	-9	-9	-9	-9	27.0	-8.0	-8.0	-8.0	-8.0	-8.0	-8	-8	-8	⋆(1-8)	[29]
Nigeria	Garki, village 202	-999.00	-99.00	-9	-9	-9	-9	5.5	-8.0	-8.0	-8.0	-8.0	-8.0	-8	-8	-8	◆(>9)	[29]
Nigeria	Garki, village 218	-999.00	-99.00	-9	-9	-9	-9	30.0	-8.0	-8.0	-8.0	-8.0	-8.0	-8	-8	-8	⋆(1-8)	[29]
Nigeria	Garki, village 218	-999.00	-99.00	-9	-9	-9	-9	7.8	-8.0	-8.0	-8.0	-8.0	-8.0	-8	-8	-8	◆(>9)	[29]
Nigeria	Garki, village 304	-999.00	-99.00	-9	-9	-9	-9	22.5	-8.0	-8.0	-8.0	-8.0	-8.0	-8	-8	-8	⋆(1-8)	[29]
Nigeria	Garki, village 304	-999.00	-99.00	-9	-9	-9	-9	7.3	-8.0	-8.0	-8.0	-8.0	-8.0	-8	-8	-8	◆(>9)	[29]
Nigeria	Garki, village 408	-999.00	-99.00	-9	-9	-9	-9	30.5	-8.0	-8.0	-8.0	-8.0	-8.0	-8	-8	-8	⋆(1-8)	[29]
Nigeria	Garki, village 408	-999.00	-99.00	-9	-9	-9	-9	6.3	-8.0	-8.0	-8.0	-8.0	-8.0	-8	-8	-8	◆(>9)	[29]
Nigeria	Garki, village 553	-999.00	-99.00	-9	-9	-9	-9	26.0	-8.0	-8.0	-8.0	-8.0	-8.0	-8	-8	-8	⋆(1-8)	[29]
Nigeria	Garki, village 553	-999.00	-99.00	-9	-9	-9	-9	4.5	-8.0	-8.0	-8.0	-8.0	-8.0	-8	-8	-8	◆(>9)	[29]
Nigeria	Garki, village 55	-999.00	-99.00	-9	-9	-9	-9	29.5	-8.0	-8.0	-8.0	-8.0	-8.0	-8	-8	-8	◆(>9)	[29]
Nigeria	Garki, village 55	-999.00	-99.00	-9	-9	-9	-9	8.8	-8.0	-8.0	-8.0	-8.0	-8.0	-8	-8	-8	◆(>9)	[29]
Nigeria	Garki, village 802	-999.00	-99.00	-9	-9	-9	-9	27.5	-8.0	-8.0	-8.0	-8.0	-8.0	-8	-8	-8	⋆(1-8)	[29]
Nigeria	Garki, village 802	-999.00	-99.00	-9	-9	-9	-9	8.3	-8.0	-8.0	-8.0	-8.0	-8.0	-8	-8	-8	◆(>9)	[29]
Nigeria	Kaduna area	-999.00	-99.00	05	1963	08	1963	26.5	-8.0	-8.0	-8.0	-8.0	8.0	-8	-8	R	-	[30]
Nigeria	Lagos	3.40	6.45[i]	-8	1949	-8	1949	11.8	-8.0	-8.0	44.8	-8.0	-8.0	-8	-8	U	⋆(0-1)	[31]
Nigeria	Lagos	3.40	6.45[i]	-8	-8	-8	-8	30.2	-8.0	-8.0	38.8	-8.0	-8.0	-8	-8	U	⋆(1-2)	[31]
Nigeria	village in Yoruba country	-999.00	-99.00	11	1951	12	1951	-8.0	-8.0	9.8	-8.0	-8.0	14.3	-8	-8	R	⋆(0-15); microscope detection	[32]
Papua New Guinea	72 villages around Madang	-999.00	-99.00	07	1981	01	1983	6.3	5.5	7.0	15.3	14.6	16.0	-8	-8	R	⋆(0-14)	[33]
Papua New Guinea	Butelgut	145.75	-5.15[i]	06	1983	09	1985	14.7	-8.0	-8.0	23.3	-8.0	-8.0	-8	-8	R	⋆(0-20)	[34]
Papua New Guinea	Mebat	145.78	-5.083	06	1983	09	1985	14.3	-8.0	-8.0	25.6	-8.0	-8.0	-8	-8	R	⋆(0-20)	[34]
Papua New Guinea	Mebat	145.78	-5.083	06	1983	09	1985	7.9	-8.0	-8.0	15.9	-8.0	-8.0	-8	-8	R	⋆(0-20)	[34]
Senegal	Aéré Lao and Boké Diallobé	-14.30	16.40[b]	05	1982	08	1983	-8.0	0.0	4.8	-8.0	0.0	28.2	R	R2	R	-	[35]
Senegal	Dakar, Grande Niaye Mrash	-17.42	14.75[r]	05	1987	09	1988	1.3	-8.0	-8.0	34.4	-8.0	-8.0	-8	U	U	◆	[36]
Senegal	Diohine	-16.51	14.48[a]	10	1996	11	1996	16.7	-8.0	-8.0	25.8	-8.0	-8.0	-8	R1	R	★(19-66)	[37]
Senegal	Diohine, Kotiokh and Ngayokhème	-999.00	-99.00	02	1995	11	1995	26.0	15.0	45.0	45.9	36.6	54.9	R	R1	R	⋆(0-9)	[38]
Senegal	Diohine, Kotiokh and Ngayokhème	-999.00	-99.00	02	1995	11	1995	9.0	3.0	20.0	26.8	18.2	30.8	R	R1	R	★	[38]
Senegal	Thies	-16.93	14.80[i]	-8	-8	-8	-8	4.8	-8.0	-8.0	53.2	-8.0	-8.0	-8	-8	R	★(>14)	[13]
Senegal	Thies	-16.93	14.80[i]	-8	-8	-8	-8	7.5	-8.0	-8.0	42.9	-8.0	-8.0	-8	-8	R	⋆(0-14)	[13]
Sierra Leone	8 villages near Bo	-999.00	-99.00	03	1990	12	1990	10.9	-8.0	-8.0	17.9	-8.0	-8.0	-8	-8	R	⋆(0.3-7)	[39]
Sudan	Asar	13.75	35.25[a]	10	1998	08	1999	29.8	12.2	52.3	-8.0	-8.0	-8.0	-8	R2	R	◆; RT-PCR detection	[40]
Sudan	Asar	13.75	35.25[a]	10	1998	08	1999	3.5	0.0	6.2	13.2	0.0	-8.0	-8	R2	R	◆; microscope detection	[40]
Sudan	Asar	-999.00	-99.00	10	1999	10	1999	-8.0	-8.0	-8.0	62.0	-8.0	-8.0	-8	R2	R	◆(6-50); RT-PCR detection	[41]
Sudan	Asar	-999.00	-99.00	10	1999	10	1999	-8.0	-8.0	-8.0	6.5	-8.0	-8.0	-8	R2	R	◆(6-50); detection by microscopy	[41]
Tanzania	40 miles west of Tanga	-999.00	-99.00	09	1952	12	1952	7.7	-8.0	-8.0	14.0	-8.0	-8.0	-8	-8	S	◆	[42]
Tanzania	Kisegese	-999.00	-99.00	-8	1992	-8	1994	11.6	-8.0	-8.0	-8.0	-8.0	-8.0	-8	-8	-8	⋆(1-19)	[23]
Tanzania	Kisegese	-999.00	-99.00	-8	1992	-8	1994	7.0	-8.0	-8.0	-8.0	-8.0	-8.0	-8	-8	-8	★(>19)	[23]
Tanzania	Northeastern Tanzania	-999.00	-99.00	-8	-8	-8	-8	13.5	-8.0	-8.0	28.0	-8.0	-8.0	-8	-8	-8	⋆(0-14); graphically derived	[24]
Tanzania	Northeastern Tanzania	-999.00	-99.00	-8	-8	-8	-8	3.7	-8.0	-8.0	11.2	-8.0	-8.0	-8	-8	-8	★(>14); graphically derived	[24]
Thailand	Ban Phluang	-999.00	-99.00	06	1983	05	1985	4.6	1.1	7.7	-8.0	-8.0	-8.0	-8	-8	R	★(>13)	[43]
Thailand	Ban Phluang	-999.00	-99.00	06	1983	05	1985	6.4	3.2	9.4	-8.0	-8.0	-8.0	-8	-8	R	⋆(1-14)	[43]
Uganda	Lira	-999.00	-99.00	09	1953	12	1953	16.9	-8.0	-8.0	28.6	-8.0	-8.0	-8	-8	S	◆; East African plateau	[44]

References

1. Hay SI, Rogers DJ, Toomer JF, Snow RW: **Annual *Plasmodium falciparum* entomological inoculation rates (EIR) across Africa: literature survey, internet access and review.** *Trans R Soc Trop Med Hyg* 2000, **94**:113–127.
2. Hay SI, Guerra CA, Tatem AJ, Atkinson PM, Snow RW: **Urbanization, malaria transmission and disease burden in Africa.** *Nat Rev Microbiol* 2005, **3**:81–90.
3. Gazin P, andand P Carnevale VR: **Le paludisme urbain à Bobo-Dioulasso (Burkina Faso). 2. Les indices paludologiques.** *Cahiers O.R.S.T.O.M. Série Entomologie Médicale et Parasitologie* 1987, **25**:27–31.
4. Gazin P, Robert V, Cot M, Simon J, Halna JM, Darriet F, Legrand D, Carnevale P, Ambroise-Thomas P: **Le paludisme dans l'Oudalan, région sahélienne du Burkina Faso.** *Ann Soc Belg Med Trop* 1988, **68**:255–264.
5. Boudin C, Robert V, Verhave JP, Carnevale P, Ambroise-Thomas P: ***Plasmodium falciparum* and *P. malariae* epidemiology in a West African village.** *Bull World Health Org* 1991, **69**:199–205.
6. Boudin C, Robert V, Carnevale P, Ambroise-Thomas P: **Epidemiology of *Plasmodium falciparum* in a rice field and a savanna area in Burkina Faso. Comparative study on the acquired immunoprotection in native populations.** *Acta Trop* 1992, **51**:103–111.
7. Boudin C, Robert V, Carnevale P, Ambroise TP: **Epidemiology of *Plasmodium falciparum* in a rice field and a savanna area in Burkina Faso: seasonal fluctuations of gametocytaemia and malaria infectivity.** *Ann Trop Med Parasitol* 1991, **85**:377–385.
8. Sabatinelli G, Bosman A, Lamizana L, Rossi P: **Prévalence du paludisme à Ouagadougou et dans le milieu rural limitrophé en periode de transmission maximale.** *Parassitologia* 1986, **28**:17–31.
9. Ouédraogo AL, Schneider P, de Kruijf M, Nébié I, Verhave JP, Cuzin-Ouattara N, Sauerwein RW: **Age-dependent distribution of *Plasmodium falciparum* gametocytes quantified by Pfs25 real-time QT-NASBA in a cross-sectional study in Burkina Faso.** *Am J Trop Med Hyg* 2007, **76**:626–630.
10. Meunier JY, Safeukui I, Fontenille D, Boudin C: **Etude de la transmission du paludisme dans une future zone d'essai vaccinal en forêt équatoriale du sud Cameroun.** *Bull Soc Pathol Exot* 1999, **92**:309–312.
11. Bonnet S, Gouagna LC, Paul RE, Safeukui I, Meunier JY, Boudin C: **Estimation of malaria transmission from humans to mosquitoes in two neighbouring villages in south Cameroon: evaluation and comparison of several indices.** *Trans R Soc Trop Med Hyg* 2003, **97**:53–59.
12. Bonnet S, Paul RE, Gouagna C, Safeukui I, Meunier JY, Gounoue R, Boudin C: **Level and dynamics of malaria transmission and morbidity in an equatorial area of South Cameroon.** *Trop Med Int Health* 2002, **7**:249–256.
13. Boudin C, Diop A, Gaye A, Gadiaga L, Gouagna C, Safeukui I, Bonnet S: ***Plasmodium falciparum* transmission blocking immunity in three areas with perennial or seasonal endemicity and different levels of transmission.** *Am J Trop Med Hyg* 2005, **73**:1090–1095.
14. Paul REL, Bonnet S, Boudin C, Tchuinkam T, Robert V: **Aggregation in malaria parasites places limits on mosquito infection rates.** *Infect Genet Evol* 2007, **7**:577–586.
15. Tchuinkam T, Mulder B, Dechering K, Stoffels H, Verhave JP, Cot M, Carnevale P, Meuwissen JHET, Robert V: **Experimental infections of *Anopheles gambiae* with *Plasmodium falciparum* of naturally infected gametocyte carriers in Cameroon: factors influencing the infectivity to mosquitoes.** *Trop Med Parasitol* 1993, **44**:271–276.
16. Mulder B, van der Ligt W, Sauerwein R, Verhave JP: **Detection of *Plasmodium falciparum* gametocytes with the QBC® test and Giemsa-stained thick blood films for malaria transmission studies in Cameroon.** *Trans R Soc Trop Med Hyg* 1998, **92**:395–396.
17. van der Kolk M, Tebo AE, Nimpaye H, Ndombol DN, Sauerwein RW, Eling WMC: **Transmission of *Plasmodium falciparum* in urban Yaoundé, Cameroon, is seasonal and age-dependent.** *Trans R Soc Trop Med Hyg* 2003, **97**:375–379.
18. Manga L, Traore O, Cot M, Mooh E, Carnevale P: **Le paludisme dans la ville de Yaoundé (Cameroon). 3. - Étude parasitologique dans deux quartiers centraux.** *Bull Soc Pathol Exot* 1993, **86**:56–61.

19. Henry MC, Rogier C, Nzeyimana I, Assi SB, Dossou-Yovo J, Audibert M, Mathonnat J, Keundjian A, Akodo E, Teuscher T, Carnevale P: **Inland valley rice production systems and malaria infection and disease in the savannah of Côte d'Ivoire.** *Trop Med Int Health* 2003, **8**:449–458.
20. Trape JF: **Études sur le paludisme dans une zone de mosaique forêt-savane d'Afrique centrale, la région de Brazzaville. II. Densités parasitaires.** *Bull Soc Pathol Exot* 1987, **80**:520–531.
21. Dunyo S, Milligan P, Edwards T, Sutherland C, Targett G, Pinder M: **Gametocytaemia after drug treatment of asymptomatic *Plasmodium falciparum*.** *PLoS Clin Trials* 2006, **1**:e20.
22. Nwakanma D, Kheir A, Sowa M, Dunyo S, Jawara M, Pinder M, Milligan P, Walliker D, Babiker HA: **High gametocyte complexity and mosquito infectivity of *Plasmodium falciparum* in The Gambia.** *Int J Parasitol* 2008, **38**:219–227.
23. Drakeley CJ, Akim NIJ, Sauerwein RW, Greenwood BM, Targett GAT: **Estimates of the infectious reservoir of *Plasmodium falciparum* malaria in The Gambia and in Tanzania.** *Trans R Soc Trop Med Hyg* 2000, **94**:472–476.
24. Drakeley C, Sutherland C, Bousema JT, Sauerwein RW, Targett GA: **The epidemiology of *Plasmodium falciparum* gametocytes: weapons of mass dispersion**. *Trends Parasitol* 2006, **22**:424–430.
25. Muirhead-Thomson RC: **Factors determining the true reservoir of infection of *Plasmodium falciparum* and *Wuchereria bancrofti* in a West African village.** *Trans R Soc Trop Med Hyg* 1954, **48**:208–225.
26. Garnham PCC: **Malarial immunity in Africans: effects in infancy and early childhood.** *Ann Trop Med Parasitol* 1949, **43**:47–61.
27. Muirhead-Thomson RC: **The malarial infectivity of an African village population to mosquitoes (*Anopheles gambiae*): a random xenodiagnostic survey.** *Am J Trop Med Hyg* 1957, **6**:971–979.
28. Miller MJ: **Observations on the natural history of malaria in the semi-resistant West African.** *Trans R Soc Trop Med Hyg* 1958, **52**:152–168.
29. Nedelman J: **Gametoctaemia and infectiousness in falciparum malaria: observations and models.** *Advances in Disease Vector Research* 1989, **6**:59–89.
30. Service MW: **Some basic entomological factors concerned with the transmission and control of malaria in northern Nigeria.** *Trans R Soc Trop Med Hyg* 1965, **59**:292–296.
31. Bruce-Chwatt LJ: **Gametocyte rates.** *Trans R Soc Trop Med Hyg* 1951, **44**:761–763.
32. Draper CC: **Observations on the infectiousness of gametocytes in hyperendemic malaria.** *Trans R Soc Trop Med Hyg* 1953, **47**:160–165.
33. Cattani JA, Tulloch JL, Vrbova H, Jolley D, Gibson FD, Moir JS, Heywood PF, Alpers MP, Stevenson A, Clancy R: **The epidemiology of malaria in a population surrounding Madang, Papua New Guinea.** *Am J Trop Med Hyg* 1986, **35**:3–15.
34. Graves PM, Burkot TR, Carter R, Cattani JA, Lagog M, Parker J, Brabin BJ, Gibson FD, Bradley DJ, Alders MP: **Measurement of malarial infectivity of human populations to mosquitoes in the Madang area, Papua New Guinea.** *Parasitology* 1988, **96**:251–263.
35. Vercruysse J: **Étude entomologique sur la transmission du paludisme humain dans le bassin du fleuve Sénégal (Senegal).** *Ann Soc Belg Med Trop* 1985, **65 (Suppl. 2)**:171–179.
36. Trape JF, Lefebvre-Zante E, Legros F, Ndiaye G, Bouganali H, Druilhe P, Salem G: **Vector density gradients and the epidemiology of urban malaria in Dakar, Senegal.** *Am J Trop Med Hyg* 1992, **47**:181–189.
37. Sokhna CS, Faye FBK, Spiegel A, Dieng H, Trape JF: **Rapid reappearance of *Plasmodium falciparum* after drug treatment among Senegalese adults exposed to moderate seasonal transmission.** *Am J Trop Med Hyg* 2001, **65**:167–17.
38. Robert V, Dieng H, Lochouam L, Traoré SF, Trape JF, Simondon F, Fontenille D: **La transmission du paludisme dans la zone de Niakhar, Sénégal.** *Trop Med Int Health* 1998, **3**:667–677.

39. Barnish G, Maude GH, Bockarie MJ, Erunkulu OA, Dumbuya MS, Greenwood BM: **Malaria in a rural area of Sierra Leone. II. Parasitological and related results from pre- and post-rains clinical surveys.** *Ann Trop Med Parasitol* 1993, **87**:137–148.
40. Abdel-Wahab A, Abdel-Muhsin AMA, Ali E, Suleiman S, Ahmed S, Walliker D, Babiker HA: **Dynamics of gametocytes among *Plasmodium falciparum* clones in natural infections in an area of highly seasonal transmission.** *J Infect Dis* 2002, **185**:1838–1842.
41. Ali E, Mackinnon MJ, Abdel-Muhsin AA, Ahmed S, Walliker D, Babiker HA: **Increased density but not prevalence of gametocytes following drug treatment of *Plasmodium falciparum*.** *Trans R Soc Trop Med Hyg* 2006, **100**:176–183.
42. Davidson G, Draper CC: **Field studies of some of the basic factors concerned in the transmission of malaria.** *Trans R Soc Trop Med Hyg* 1953, **47**:522–535.
43. Rosenberg R, Andre RG, Somchit L: **Highly efficient dry season transmission of malaria in Thailand.** *Trans R Soc Trop Med Hyg* 1990, **84**:22–28.
44. Davidson G: **Further studies of the basic factors concerned in the transmission of malaria.** *Trans R Soc Trop Med Hyg* 1955, **49**:339–350.
