# Supplementary material for: Development of a new version of the Liverpool Malaria Model. I. Refining the parameter settings and mathematical formulation of basic processes based on a literature review
Source: Malar J. 2011 Feb 11;10:35. doi: 10.1186/1475-2875-10-35 (PMC3055220; doi:10.1186/1475-2875-10-35)
Supplement: Additional file 7 — Human-to-mosquito transmission efficiencies. Data with regard to the human-to-mosquito transmission efficiency (c), i.e. the proportion of mosquito bites on infectious humans which infect susceptible mosquitoes. [file 1475-2875-10-35-S7.PDF]

7 Human-to-mosquito transmission efficiencies

Data with regard to the human-to-mosquito transmission efficiency (c) that is the proportion of mosquito bites on infectious humans which infect susceptible mosquitoes.

Columns: country: country where the study was undertaken; place: location of the study site; long: longitude of the study site (-999.00: position is either unknown or was not seeked out); lat: latitude of the study site (-99.00: position is either unknown or was not seeked out); M1: month, when the study started; YYY1: year of the start of the study; M2: month, when the study ended; YYY2: year of the end of the study; c human-to-mosquito transmission efficiency [%]; U_b: land use classification as defined by Hay *et al.* [1]: R=rural, I=irrigated rice, U= urban area; U_a: as U_b but after Hay *et al.* [2]: PU=peri-urban (population densities of 250-1,000 persons per km^2), R1=rural 1 (population densities of 100-250 persons per km^2), R2=rural 2 (population densities of <100 persons per km^2), U=urban (population densities of $>1{,}000$ persons per km^2); U_p: as U_b, but as taken from the particular reference: R=rural area, I=irrigation/permanent stream or river, U=urban area; notes: notes; ref: reference. The '-8' and '-9' denote data that were not available in the literature and that could not be checked due to limited access, respectively. Indices: ^{i}: the position of the study site was taken from http://www.heavens-above.com/countries.aspx; ^{m}: the position of the study site was derived from a published map; ^{r}: the position of the study site was found in the reference. Symbols: ⋆: children (the following numbers indicate age classes in years); ★: adults; ◆: all ages.

country	place	long [°E]	lat [°N]	M1	YYY1	M2	YYY2	c	U_b [1]	U_a [2]	U_p	notes	ref
Burkina Faso	near Bobo-Dioulasso	-999.00	-99.00	-8	1985	-8	1987	37.2	R	R2	R	◆; experimental feeding on heavy carriers of gametocytes	[3]
Cameroon	Bondi	12.19	3.86^m	-8	1998	-8	2000	11.8	-8	-8	R	★(>14); area: degraded forest	[4]
Cameroon	Bondi	12.19	3.86^m	-8	1998	-8	2000	36.9	-8	-8	R	⋆(0-14); area: degraded forest	[4]
Cameroon	district of Mengang	-999.00	-99.00	-8	-8	-8	-8	12.1	-8	-8	R	◆(>5); membran feeding	[5]
Cameroon	district of Mengang	-999.00	-99.00	-8	-8	-8	-8	19.4	-8	-8	R	◆(>5); direct skin feeding	[5]
Cameroon	Mengang	12.05	3.88^i	-8	1998	-8	2000	15.5	-8	-8	R	⋆(0-14); area: degraded forest	[4]
Cameroon	Mengang	12.05	3.88^i	-8	1998	-8	2000	7.7	-8	-8	R	★(>14); area: degraded forest	[4]
Cameroon	Mengang district	-999.00	-99.00	-8	-8	-8	-8	33.0	-8	-8	R	◆(>4)	[6]
Cameroon	Yaoundé, Messa quarter	-999.00	-99.00	10	1990	01	1993	18.6	-8	-8	U	◆(4-60)	[7]
Cameroon	Yaoundé, Messa quarter	-999.00	-99.00	-8	-8	-8	-8	12.8	-8	-8	U	membran feeding with blood from 65 gametocyte carriers; ◆(6-36)	[8]
Cameroon	Yaoundé, Messa quarter	-999.00	-99.00	-8	-8	-8	-8	12.8	-8	-8	U	membran feeding with blood from 65 gametocyte carriers; ◆(6-36)	[8]
Cameroon	Yaounde, urban district	-999.00	-99.00	-8	-8	-8	-8	20.0	-8	-8	U	◆(>4)	[6]
Gambia, The	5 villages around Farafenni	-16.57	13.47^r	05	2003	10	2003	43.0	-8	-8	R	membrane feedings on blood following drug treatment	[9]
Gambia, The	5 villages around Farafenni	-16.57	13.47^r	05	2003	10	2003	6.4	-8	-8	R	47 trials of membran feeding on gametocyte carriers; ◆(5-45)	[9]
Gambia, The	near Farafenni, north bank villages	-999.00	-99.00	-8	-8	-8	-8	56.5	-8	-8	-8	⋆(1-19)	[10]
Gambia, The	near Farafenni, north bank villages	-999.00	-99.00	-8	-8	-8	-8	80.0	-8	-8	-8	★(>19)	[10]
Ghana	-9	-999.00	-99.00	01	1952	10	1952	0.0	-8	-8	-9	★(>14) (two)	[11]
Ghana	-9	-999.00	-99.00	01	1952	10	1952	30.0	-8	-8	-9	⋆(0-14) (40)	[11]
Ghana	Accra	-999.00	-99.00	-8	-8	-8	-8	50.9	-8	-8	U	◆	[12]
Ghana	Weija	-999.00	-99.00	01	1952	10	1952	26.6	-8	-8	R	⋆(0.8-10)	[11]
Liberia	-9	-999.00	-99.00	-9	-9	-9	-9	20.1	-8	-8	-9	★(>14)	[13]
Liberia	-9	-999.00	-99.00	-9	-9	-9	-9	21.6	-8	-8	-9	⋆(0-14)	[13]
Liberia	village	-999.00	-99.00	12	1955	11	1956	19.1	-8	-8	R	⋆(0-4)	[13]
Liberia	village	-999.00	-99.00	12	1955	11	1956	20.1	-8	-8	R	⋆(>14)	[13]
Liberia	village	-999.00	-99.00	12	1955	11	1956	23.3	-8	-8	R	⋆(5-14)	[13]
Nigeria	Kaduna area	-999.00	-99.00	05	1963	08	1963	20.0	-8	-8	R	low gametocyte density	[14]
Nigeria	Kaduna area	-999.00	-99.00	05	1963	08	1963	50.0	-8	-8	R	high gametocyte density	[14]
Nigeria	Lagos	-999.00	-99.00	-8	-8	-8	-8	46.6	-8	-8	U	⋆	[12]

to be continued

Table 1 – continued

country	place	long [°E]	lat [°N]	M1	YYY1	M2	YYY2	c	U_b [1]	U_a [2]	U_p	notes	ref
Nigeria	village in Yoruba country	-999.00	-99.00	11	1951	12	1951	9.0	-8	-8	R	⋆(0-10)	[12]
Papua New Guinea	Buksak	-999.00	-99.00	02	1986	12	1986	48.1	-8	-8	R	-	[15]
Papua New Guinea	Butelgut, Mebat, Sah, Buksak	-999.00	-99.00	-8	1985	-8	1985	37.9	-8	-8	R	◆	[16]
Senegal	Thies	-16.93	14.80^{i}	-8	-8	-8	-8	25.0	-8	-8	R	◆(>4)	[6]
Tanzania	Kisegese	-999.00	-99.00	-8	-8	-8	-8	26.7	-8	-8	-8	★(>19)	[10]
Tanzania	Kisegese	-999.00	-99.00	-8	-8	-8	-8	44.1	-8	-8	-8	⋆(1-19)	[10]
Thailand	Phra Phutthabat	100.8	14.72^{i}	06	1965	10	1967	23.2	-8	-8	-8	◆(12-60); rainy season	[17]
Thailand	Phra Phutthabat	100.8	14.72^{i}	06	1965	10	1967	37.9	-8	-8	-8	◆(12-60); cool season	[17]
Thailand	Phra Phutthabat	100.8	14.72^{i}	06	1965	10	1967	9.7	-8	-8	-8	◆(12-60); hot season	[17]

References

1. Hay SI, Rogers DJ, Toomer JF, Snow RW: **Annual *Plasmodium falciparum* entomological inoculation rates (EIR) across Africa: literature survey, internet access and review.** *Trans R Soc Trop Med Hyg* 2000, **94**:113–127.
2. Hay SI, Guerra CA, Tatem AJ, Atkinson PM, Snow RW: **Urbanization, malaria transmission and disease burden in Africa.** *Nat Rev Microbiol* 2005, **3**:81–90.
3. Boudin C, Robert V, Carnevale P, Ambroise TP: **Epidemiology of *Plasmodium falciparum* in a rice field and a savanna area in Burkina Faso: seasonal fluctuations of gametocytaemia and malaria infectivity.** *Ann Trop Med Parasitol* 1991, **85**:377–385.
4. Bonnet S, Gouagna LC, Paul RE, Safeukui I, Meunier JY, Boudin C: **Estimation of malaria transmission from humans to mosquitoes in two neighbouring villages in south Cameroon: evaluation and comparison of several indices.** *Trans R Soc Trop Med Hyg* 2003, **97**:53–59.
5. Bonnet S, Paul RE, Gouagna C, Safeukui I, Meunier JY, Gounoue R, Boudin C: **Level and dynamics of malaria transmission and morbidity in an equatorial area of South Cameroon.** *Trop Med Int Health* 2002, **7**:249–256.
6. Boudin C, Diop A, Gaye A, Gadiaga L, Gouagna C, Safeukui I, Bonnet S: ***Plasmodium falciparum* transmission blocking immunity in three areas with perennial or seasonal endemicity and different levels of transmission.** *Am J Trop Med Hyg* 2005, **73**:1090–1095.
7. Tchuinkam T, Mulder B, Dechering K, Stoffels H, Verhave JP, Cot M, Carnevale P, Meuwissen JHET, Robert V: **Experimental infections of *Anopheles gambiae* with *Plasmodium falciparum* of naturally infected gametocyte carriers in Cameroon: factors influencing the infectivity to mosquitoes.** *Trop Med Parasitol* 1993, **44**:271–276.
8. Mulder B, Tchuinkam T, Dechering K, Verhave JP, Carnevale P, Meuwissen JHET, Robert V: **Malaria transmission-blocking activity in experimental infections of *Anopheles gambiae* from naturally infected *Plasmodium falciparum* gametocyte carriers.** *Trans R Soc Trop Med Hyg* 1994, **88**:121–125.
9. Nwakanma D, Kheir A, Sowa M, Dunyo S, Jawara M, Pinder M, Milligan P, Walliker D, Babiker HA: **High gametocyte complexity and mosquito infectivity of *Plasmodium falciparum* in The Gambia.** *Int J Parasitol* 2008, **38**:219–227.
10. Drakeley CJ, Akim NIJ, Sauerwein RW, Greenwood BM, Targett GAT: **Estimates of the infectious reservoir of *Plasmodium falciparum* malaria in The Gambia and in Tanzania.** *Trans R Soc Trop Med Hyg* 2000, **94**:472–476.
11. Muirhead-Thomson RC: **Factors determining the true reservoir of infection of *Plasmodium falciparum* and *Wuchereria bancrofti* in a West African village.** *Trans R Soc Trop Med Hyg* 1954, **48**:208–225.
12. Draper CC: **Observations on the infectiousness of gametocytes in hyperendemic malaria.** *Trans R Soc Trop Med Hyg* 1953, **47**:160–165.
13. Muirhead-Thomson RC: **The malarial infectivity of an African village population to mosquitoes (*Anopheles gambiae*): a random xenodiagnostic survey.** *Am J Trop Med Hyg* 1957, **6**:971–979.

14. Service MW: **Some basic entomological factors concerned with the transmission and control of malaria in northern Nigeria.** *Trans R Soc Trop Med Hyg* 1965, **59**:292–296.
15. Burkot TR, Graves PM, Paru R, Battistutta D, Barnes A, Saul A: **Variations in malaria transmission rates are not related to anophelines survivorship per feeding cycle.** *Am J Trop Med Hyg* 1990, **43**:321–327.
16. Graves PM, Burkot TR, Carter R, Cattani JA, Lagog M, Parker J, Brabin BJ, Gibson FD, Bradley DJ, Alders MP: **Measurement of malarial infectivity of human populations to mosquitoes in the Madang area, Papua New Guinea.** *Parasitology* 1988, **96**:251–263.
17. Rutledge LC, Gould DJ, Tantichareon B: **Factors affecting the infection of anophelines with human malaria in Thailand.** *Trans R Soc Trop Med Hyg* 1969, **63**:613–619.
